# HbA1c and pulmonary function in type 2 diabetes mellitus: A systematic review and meta-analysis

**DOI:** 10.1371/journal.pone.0355628

**Published:** 2026-08-13

**Authors:** Kadek Surya Atmaja, Sri Masyeni, Saraswati Laksmi Dewi, Previyanti Dharma Putri

**Affiliations:** 1 Department of Internal Medicine/Pulmonology, Faculty of Medicine and Health Science, Warmadewa University, Denpasar-Bali, Indonesia; 2 Department of Internal Medicine, Faculty of Medicine and Health Science, Warmadewa University, Denpasar-Bali, Indonesia; 3 Department of Cardiology, Faculty of Medicine and Health Science, Warmadewa University, Denpasar-Bali, Indonesia; Christian Medical College, INDIA

## Abstract

**Background:**

The lung, although not traditionally been considered a target organ in diabetes, may undergo microvascular and connective tissue changes that are related to poor glycemic control. This systematic review and meta-analysis aimed to systematically evaluate and quantify the association between HbA1c values and pulmonary function in adults with type 2 diabetes mellitus.

**Methods:**

A systematic review was conducted in accordance with the PRISMA 2020 guidelines. Studies were searched in PubMed/MEDLINE, Scopus, and Web of Science Core Collection from database inception to 25 December 2025. Eligible studies compared spirometric outcomes between adults with type 2 diabetes categorized as having good versus poor glycemic control based on HbA1c. Random-effects meta-analyses were conducted using mean differences.

**Results:**

Individuals with poor glycemic control had significantly lower absolute spirometric volumes. FEV1 was lower in the poor HbA1c group, with a pooled mean difference of –0.16 L (95% CI –0.25 to –0.07; p < 0.001). Similarly, FVC was reduced, with a pooled mean difference of –0.24 L (95% CI –0.35 to –0.14; p < 0.001). In contrast, the FEV1/FVC ratio was slightly higher in the poor-control group (MD = 1.61 percentage points, 95% CI 0.50 to 2.73; p = 0.005). All pooled analyses showed no evidence of statistical heterogeneity (I^2^ = 0%).

**Conclusion:**

Poor glycemic control in T2DM is associated with lower absolute FEV1 and FVC. The preserved or slightly higher FEV1/FVC ratio suggests reduced spirometric volumes rather than definite obstructive impairment. These findings should be interpreted as group-level associations because the analyses were based on absolute spirometric values and observational studies.

## Introduction

Type 2 diabetes mellitus (T2DM) is a major global health burden affecting more than 500 million individuals worldwide, with its prevalence continuing to rise across both high- and low-income settings [1]. Chronic hyperglycemia is known to contribute to a wide spectrum of microvascular and macrovascular complications, including nephropathy, retinopathy, neuropathy, and cardiovascular disease [2]. Although the lung has historically not been viewed as a major target organ in diabetes, emerging evidence suggests that pulmonary tissue may undergo structural and functional alterations that are analogous to those observed in other diabetic complications [3,4].

The lung possesses an extensive microvascular network and is rich in connective tissue, making it particularly vulnerable to metabolic and inflammatory disturbances associated with diabetes. Experimental and clinical studies have shown that chronic hyperglycemia promotes non-enzymatic glycation of collagen and elastin, leads to the thickening of the alveolar-capillary basement membrane, and results in the accumulation of advanced glycation end-products (AGEs), all of which contribute to reduced lung compliance and impaired gas exchange [5,6]. In addition, low-grade systemic inflammation, oxidative stress, and pulmonary microangiopathy, which are frequently described in poorly controlled diabetes, may further accelerate structural changes in the lung parenchyma and contribute to progressive loss of pulmonary function [7,8].

Several observational studies have suggested that individuals with higher HbA1c levels exhibit lower FEV1 and FVC, suggesting reduced spirometric volumes and possible restrictive physiology. However, true restrictive ventilatory impairment requires confirmation using lung volume measurements, particularly total lung capacity, potentially reflecting the concept of the “diabetic lung” [9,10]. However, findings across individual studies have been inconsistent, with variations in sample size, population characteristics, and methodological rigor that limit the ability to draw firm conclusions. Specifically, many studies have lacked the statistical power to detect clinically meaningful differences, while others have used heterogeneous definitions of glycemic control or did not adequately adjust for important confounders such as age, sex, height, body weight, body mass index, race or ethnicity, smoking status, physical activity, diet or nutritional status, muscle mass, and diabetes duration.

Despite an increasing number of publications exploring this relationship, no previous meta-analysis has quantitatively synthesized the magnitude of lung function differences specifically between patients with good versus poor glycemic control using absolute, directly measured spirometric values, limiting cross-study comparability and clinical interpretability. Consequently, the extent to which chronic hyperglycemia impairs pulmonary mechanics remains uncertain.

Given these gaps, a comprehensive meta-analysis is warranted to clarify the association between HbA1c levels and pulmonary function in T2DM. Quantifying the pooled effects on FEV₁ and FVC may help establish the lung as a clinically relevant target organ in diabetes, provide insight into subclinical pulmonary involvement, and support the incorporation of respiratory assessment into routine diabetes management.

## Methods

### Study design

This study was conducted as a systematic review and meta-analysis, and was structured according to the PRISMA 2020 guidelines [11]. The methodological framework was developed a priori, clearly outlining the objectives, eligibility criteria, data extraction strategy, analytical plan, and risk-of-bias assessment. The primary aim was to quantitatively synthesize the association between glycemic control, as measured by glycated hemoglobin (HbA1c), and pulmonary function. FEV₁ and FVC were analyzed as absolute volumes expressed in liters, whereas the FEV₁/FVC ratio was harmonized and analyzed in percentage points.

### Eligibility criteria

Eligibility criteria were established a priori and applied consistently across all screening stages. Studies were included if they enrolled adult participants with confirmed T2DM and reported spirometric outcomes obtained through standardized testing procedures. Eligible studies were required to categorize participants into good versus poor glycemic control, typically based on HbA1c thresholds defined within each study. Only those providing extractable quantitative data, specifically mean and standard deviation values for spirometric parameters in each glycemic control group were considered. Observational designs, such as cross-sectional assessments or baseline data from cohort studies, were eligible for inclusion. Studies were excluded if they involved type 1 diabetes, reported only percent-predicted values without corresponding absolute measurements, failed to stratify outcomes by HbA1c status, or were non-original publications such as reviews, letters, or conference abstracts.

### Information sources and search strategy

PubMed/MEDLINE, Scopus, and Web of Science Core Collection were searched from database inception to 25 December 2025. The search strategy was structured around three core concepts: type 2 diabetes mellitus, glycemic control assessed using HbA1c, and pulmonary function or spirometry. Controlled vocabulary terms, including Medical Subject Headings (MeSH), were combined with free-text keywords, synonyms, and relevant spelling variants using the Boolean operators “AND” and “OR.”

A representative PubMed search strategy was: (“Diabetes Mellitus, Type 2”[Mesh] OR “type 2 diabetes”[Title/Abstract] OR “type II diabetes”[Title/Abstract] OR T2DM[Title/Abstract]) AND (“Hemoglobin A, Glycosylated”[Mesh] OR HbA1c[Title/Abstract] OR “glycated hemoglobin”[Title/Abstract] OR “glycated haemoglobin”[Title/Abstract] OR “glycemic control”[Title/Abstract] OR “glycaemic control”[Title/Abstract]) AND (“Pulmonary Function Tests”[Mesh] OR “pulmonary function”[Title/Abstract] OR “lung function”[Title/Abstract] OR spirometry[Title/Abstract] OR “forced expiratory volume”[Title/Abstract] OR FEV1[Title/Abstract] OR “forced vital capacity”[Title/Abstract] OR FVC[Title/Abstract]). Complete database-specific search strategies for PubMed/MEDLINE, Scopus, and Web of Science are provided in Supplementary S1 Appendix. Reference lists of included studies and relevant review articles were also screened manually to identify additional eligible studies.

### Study selection

Study selection was carried out by three independent reviewers, who performed blinded screening using the Rayyan web-based platform [12]. Rayyan facilitated efficient duplicate removal, labeling, and conflict detection throughout the screening process. Initially, titles and abstracts were reviewed to identify studies that potentially met the inclusion criteria. Articles deemed relevant were subjected to full-text assessment to determine final eligibility. Any conflicts in study selection were resolved through discussion among the reviewers until consensus was achieved. The selection process adhered to the PRISMA guidelines and comprised four distinct phases: identification, screening, eligibility assessment, and final inclusion for meta-analysis.

### Data extraction

Data extraction was conducted independently by the three reviewers using a standardized extraction form. Extracted information included study characteristics (author, year, country, design), sample size, participant demographics, definitions of glycemic control categories, and the spirometric outcomes FEV₁, FVC, and FEV₁/FVC reported as mean ± standard deviation. When available, additional variables relevant to potential confounding were also extracted, including age, sex, height, body weight, body mass index, smoking status, race or ethnicity, duration of diabetes, physical activity, diet or nutritional status, and muscle mass. These variables were recorded to assess clinical comparability across studies and to contextualize the interpretation of pooled estimates. However, because these factors were inconsistently reported and adjusted for across the primary studies, they could not be uniformly incorporated into the quantitative synthesis. All discrepancies identified during the extraction process were resolved through discussion to ensure the completeness and accuracy of the final dataset used for quantitative synthesis.

### Risk of bias assessment

Each included study was critically appraised using the original eight-item Joanna Briggs Institute (JBI) Critical Appraisal Checklist for Analytical Cross-Sectional Studies [13]. The checklist was applied without modification; no items were merged, omitted, weighted, or converted into a numerical score. The eight items assessed: [1] clarity of the inclusion criteria; [2] detailed description of the study participants and setting; [3] validity and reliability of exposure measurement; [4] use of objective and standard criteria for measurement of the condition; [5] identification of potential confounding factors; [6] strategies used to address confounding factors; [7] validity and reliability of outcome measurement; and [8] appropriateness of the statistical analysis. Each item was rated as “Yes,” “No,” “Unclear,” or “Not applicable” in accordance with the original JBI guidance. Overall appraisal was recorded using the JBI categories “Include,” “Exclude,” or “Seek further information.” The assessment was performed independently by three reviewers, and disagreements were resolved through discussion and consensus. Item-level judgments for all included studies are presented in Supplementary S5 Appendix.

### Statistical analysis

A random-effects meta-analysis was performed to synthesize effect estimates across studies, accounting for anticipated heterogeneity in study populations and methodologies. For FEV1 and FVC, pooled mean differences (MDs) were calculated using absolute spirometric values reported in liters. For the FEV1/FVC ratio, all values were harmonized into percentage points and pooled using mean differences to preserve clinical interpretability.

When studies reported more than two HbA1c categories, categories above the prespecified good-control threshold were combined into a single poor-control group using standard formulas for pooled means and standard deviations. Meta-analyses were rerun using Stata [14] to improve reproducibility and to facilitate transparent handling of studies requiring category combination. Random-effects models using the DerSimonian–Laird method were applied. Statistical heterogeneity was evaluated using Cochran’s Q test and quantified with the I^2^ statistic. Certainty of Evidence Assessment.

The certainty of evidence for each outcome was assessed using the Grading of Recommendations Assessment, Development and Evaluation (GRADE) approach [15]. As all included studies were observational in design, the initial certainty of evidence was rated as low. The certainty was then evaluated across five domains: risk of bias, inconsistency, indirectness, imprecision, and publication bias. Evidence was downgraded or upgraded based on predefined GRADE criteria. Assessments were performed independently by reviewers, and final judgments were reached by consensus. The overall certainty of evidence for each outcome was classified as high, moderate, low, or very low.

## Result

### Study selection

**Fig 1:**
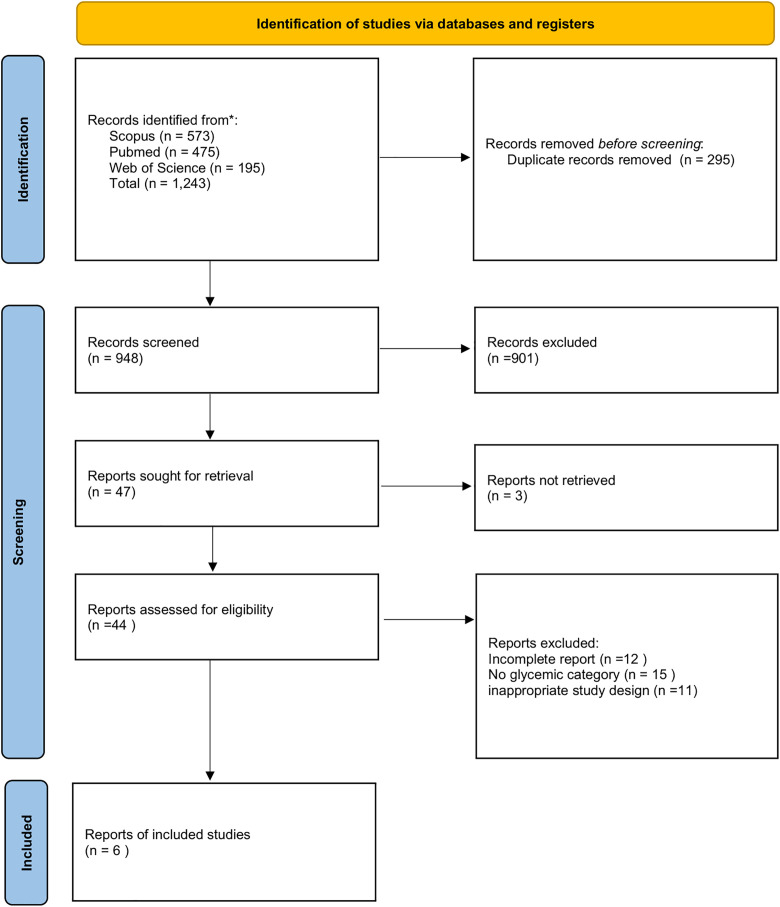
PRISMA 2020 flow diagram. Diagram illustrating the identification, screening, eligibility assessment, and final inclusion of studies in the systematic review.

The database search identified 1,243 records from Scopus, PubMed, and Web of Science. After removal of 295 duplicate records, 948 records were screened by title and abstract. Of these, 901 records were excluded, and 47 reports were sought for retrieval. Three reports could not be retrieved, leaving 44 full-text reports assessed for eligibility. Thirty-eight reports were excluded for the following reasons: incomplete reporting of spirometric data (n = 12), absence of glycemic control categories (n = 15), and inappropriate study design (n = 11). Ultimately, six studies met the eligibility criteria and were included in the quantitative synthesis. The PRISMA flow diagram is presented in Fig 1.

### Study characteristics

The six included studies encompassed 944 adults with type 2 diabetes mellitus from India, Saudi Arabia, and Colombia. All included studies were observational in design, primarily cross-sectional, and evaluated pulmonary function using spirometric parameters. Glycemic control was categorized according to study-specific HbA1c thresholds, ranging from 7.0% to 8.0%. All six studies, comprising 944 participants, provided extractable data for FEV1 and FVC. Five studies, comprising 801 participants, provided extractable data for the FEV1/FVC ratio. Barik et al. was the only included study that did not provide extractable FEV1/FVC ratio data for the pooled analysis. The characteristics of the included studies are summarized in Table 1, and the number of studies and participants contributing to each pooled outcome is presented in Table 2.

**Table 1:** Characteristics of the Included Studies.

Author (Year)	Country	Sample Size	HbA1c Cutoff	Spirometry Outcomes
Anandhalakshmi et al. (2013)	India	30	<7 vs>7	FEV₁, FVC, FEV₁/FVC
Maan et al. (2021)	Saudi Arabia	101	≤8 vs > 8	FEV₁, FVC, FEV₁/FVC
Singh et al. (2023)	India	120	<7 vs ≥ 7	FEV₁, FVC, FEV₁/FVC
Senthilnathan et al. (2016)	India	55	<7 vs ≥ 7	FEV₁, FVC, FEV₁/FVC
Barik et al. (2025)	India	143	<7 vs ≥ 7	FEV₁, FVC
Dennis et al. (2010)	Colombia	495	≤7 vs > 7	FEV1, FVC, FEV1/FVC

**Table 2:** Number of Studies and Participants Contributing to Each Pooled Outcome.

Outcome	Number of Studies	Total Participants	Studies Included
FEV1	6	944	Anandhalakshmi, Maan, Singh, Senthilnathan, Barik, Dennis
FVC	6	944	Anandhalakshmi, Maan, Singh, Senthilnathan, Barik, Dennis
FEV1/FVC ratio	5	801	Anandhalakshmi, Maan, Singh, Senthilnathan, Dennis

### Risk of bias assessment

The methodological quality of the six included studies was assessed using the original eight-item JBI Critical Appraisal Checklist for Analytical Cross-Sectional Studies. Overall, the included studies clearly defined their eligibility criteria and generally used valid and reliable methods for measuring HbA1c and spirometric outcomes. The principal methodological concerns involved incomplete identification of potential confounding factors and insufficiently described strategies for addressing confounding in Anandhalakshmi et al. (2013), Singh et al. (2023), and Senthilnathan et al. (2016). Relevant confounders included age, sex, height, body mass index, smoking status, duration of diabetes, and other factors known to influence pulmonary function.

Maan et al. (2021), Barik et al. (2025), and Dennis et al. (2010) met all eight JBI checklist criteria. All six studies received an overall appraisal of “Include,” as none had methodological limitations considered sufficiently serious to warrant exclusion from the review. Nevertheless, the item-level concerns relating to confounding were considered when assessing the certainty of evidence and interpreting the pooled findings. The complete eight-item appraisal for each study is provided in Supplementary S5 Appendix.

### Meta-analysis of FEV₁

**Fig 2:**
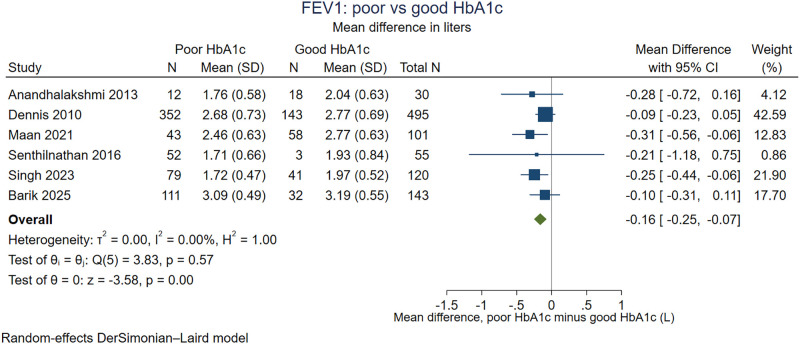
Pooled mean differences (MD) for FEV₁ between groups with poor and good glycemic control. Negative MD values indicate lower FEV₁ values among individuals with poor glycemic control.

Six studies comprising 944 participants contributed data to the meta-analysis of FEV1. Using a random-effects DerSimonian–Laird model, poor glycemic control was associated with significantly lower FEV1 compared with good glycemic control (MD = –0.16 L, 95% CI –0.25 to –0.07; p < 0.001). There was no evidence of statistical heterogeneity across studies (I² = 0.0%). These findings suggest a consistent association between poor glycemic control and lower absolute FEV1, but they should be interpreted as group-level differences rather than definitive evidence of clinically abnormal lung function (16.21). Forest plot meta-analysis of FEV1 is presented in Fig 2.

### Meta-analysis of FVC

**Fig 3:**
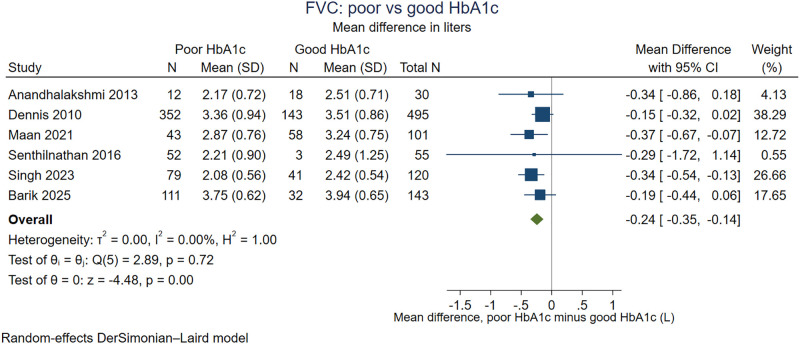
Pooled mean differences (MD) for FVC between poor and good HbA1c groups. Negative MD values indicate reduced FVC among individuals with poor glycemic control.

Six studies comprising 944 participants contributed data to the meta-analysis of FVC. Using a random-effects DerSimonian–Laird model, poor glycemic control was associated with significantly lower FVC compared with good glycemic control (MD = –0.24 L, 95% CI –0.35 to –0.14; p < 0.001). There was no evidence of statistical heterogeneity across studies (I^2^ = 0.0%). These findings indicate lower absolute FVC values among individuals with poor glycemic control. However, because the analysis was based on absolute values rather than percent-predicted values or lung volume measurements, the finding should not be interpreted as definitive evidence of true restrictive ventilatory impairment [16–21]. Forest plot metanalysis of FVC is presented in Fig 3.

### Meta-analysis of FEV₁/FVC Ratio

Five studies comprising 801 participants contributed data to the meta-analysis of the FEV1/FVC ratio. After harmonizing all reported values into percentage points, the pooled mean difference showed a small but statistically significant increase in the FEV1/FVC ratio among participants with poor glycemic control compared with those with good glycemic control (MD = 1.61 percentage points, 95% CI 0.50 to 2.73; p < 0.01), with no evidence of statistical heterogeneity (I^2^= 0.0%).

**Fig 4:**
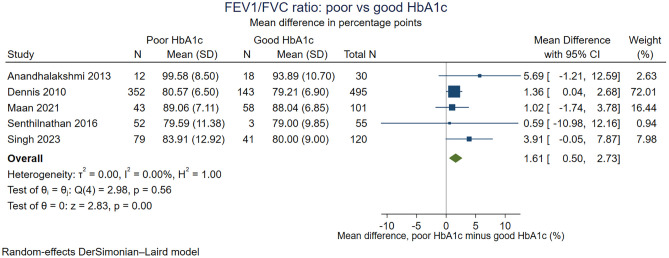
Pooled mean differences (MD) in FEV1/FVC ratio between poor and good HbA1c groups. Values were harmonized into percentage points. Positive MD values indicate higher FEV1/FVC ratio among individuals with poor glycemic control.

This finding indicates that the reductions in FEV1 and FVC were not accompanied by a reduction in the FEV1/FVC ratio. A preserved or slightly higher FEV1/FVC ratio may be more compatible with lower spirometric volumes suggestive of possible restrictive physiology rather than obstructive impairment. However, true restriction cannot be confirmed without lung volume assessment, particularly total lung capacity. Forest plot metanalysis of FEV/FVC is presented in Fig 4. Planned subgroup analyses, meta-regression, and sensitivity analyses were not conducted due to the limited number of included studies

### Publication bias

Visual inspection of the funnel plots did not show obvious asymmetry. However, because only five to six studies contributed to each outcome, funnel-plot assessment was exploratory and had limited ability to detect small-study effects. Formal statistical tests for funnel-plot asymmetry were therefore not performed, and publication bias could not be excluded. The funnel plots should be interpreted with caution and are presented for exploratory purposes. Detailed funnel plots for each outcome are provided in Supplementary Data S4.

### Certainty of evidence assessment

Overall, the certainty of evidence for FEV1, FVC, and the FEV1/FVC ratio was rated as low. All included studies were observational and therefore started at low certainty. No outcome was upgraded because there was no prespecified evidence of a large effect, a dose-response gradient, or residual confounding that would be expected to increase the observed association. Although statistical heterogeneity was negligible and the pooled estimates wee reasonably precise, incomplete adjustment for important confounders and the limited number of studies restricted confidence in the findings. Therefore, the pooled estimates should be interpreted as low-certainty evidence of group-level associations.

## Discussion

This systematic review and meta-analysis demonstrates that poor glycemic control in individuals with type 2 diabetes mellitus is associated with lower absolute FEV1 and FVC. The pooled findings showed significant reductions of –0.16 L for FEV1 and –0.24 L for FVC among individuals with poor HbA1c control. In contrast, the FEV1/FVC ratio was preserved or slightly increased, suggesting that the reduction in spirometric volumes was not accompanied by a proportional reduction in the ratio. These findings may be compatible with lower spirometric volumes suggestive of possible restrictive physiology, but true restrictive ventilatory impairment cannot be confirmed without lung volume assessment.The pathophysiology underlying diabetes-associated pulmonary impairment is multifactorial. One central mechanism is the non-enzymatic glycation of lung collagen and elastin, leading to the accumulation of advanced glycation end-products (AGEs). These molecules promote collagen cross-linking, resulting in increased stiffness of lung tissue and reduced compliance [5,6]. Thickening of the alveolar-capillary basement membrane has also been documented in autopsy specimens of diabetic patients, further supporting a structural explanation for reduced lung volumes [5].

Additionally, pulmonary microangiopathy mirrors the microvascular damage seen in diabetic nephropathy and retinopathy. Studies have shown the thickening of the alveolar-capirally walls, endothelial dysfunction, and reduced diffusing capacity in individuals with poor metabolic control [4,8]. These microvascular alterations may impair both gas exchange and lung mechanics, which may help explain why both FEV₁ and FVC are reduced.

Chronic low-grade systemic inflammation, a hallmark of poorly controlled diabetes, may also contribute to pulmonary dysfunction. Higher levels of inflammatory markers, particularly C-reactive protein, have been associated with reduced lung function and subsequent decline in FEV₁ and FVC, suggesting that inflammatory pathways may contribute to impaired pulmonary mechanics [7]. Oxidative stress also plays an important role; hyperglycemia increases the production of reactive oxygen species, causing injury to alveolar epithelial cells and promoting extracellular matrix remodeling [8].

Neuromuscular pathways may also contribute. Autonomic neuropathy, common in long-standing diabetes, has been linked to abnormalities in respiratory muscle performance and irregularities in airway tone [22]. Taken together, microvascular remodeling, AGE accumulation, oxidative stress, systemic inflammation, and autonomic dysfunction provide biological plausibility for the lower spirometric volumes observed in the present analysis.

Large population-based studies support these findings. In the Atherosclerosis Risk in Communities (ARIC) cohort, higher HbA1c was associated with an accelerated longitudinal decline in FVC and FEV₁ [3,9]. Similar results were reported in Asian cohorts, with poor glycemic control predicting reduced ventilatory capacity independently of smoking or BMI [10,23]. The present findings align closely with this evidence, suggesting that pulmonary impairment may arise early in the diabetes trajectory, even before the onset of clinically apparent microvascular complications.

The cumulative evidence suggests that these findings support awareness of possible pulmonary involvement in patients with poorly controlled or long-standing type 2 diabetes, particularly when respiratory symptoms or additional cardiometabolic risk factors are present. However, the available evidence does not support routine spirometric screening for all patients with type 2 diabetes.Given the observed association between poor glycemic control and lower lung volumes, pulmonary function assessment may be considered in selected patients with type 2 diabetes, particularly those with longstanding disease, poor metabolic control, respiratory symptoms, or multiple cardiometabolic risk factors. However, because the available evidence is derived mainly from observational studies and may be influenced by residual confounding, these findings should be interpreted as hypothesis-generating rather than as definitive evidence for routine spirometric screening in all patients with type 2 diabetes.

### Strengths and Limitations of the Study

This meta-analysis has several notable strengths. First, it synthesizes available evidence using standardized spirometric parameters across multiple independent studies, resulting in highly consistent pooled estimates with negligible heterogeneity. Second, the review integrates well-established biological mechanisms—such as extracellular matrix glycation, pulmonary microangiopathy, oxidative stress, and systemic inflammation—to reinforce the physiological plausibility of the observed reductions in FEV₁ and FVC among individuals with poor glycemic control. Third, the study offers novelty by providing one of the first pooled quantitative estimates specifically comparing actual spirometric volumes between good and poor HbA1c groups, thereby establishing a clearer magnitude of lung function deficit attributable to chronic hyperglycemia. This addresses an important gap in the existing literature, where findings have been largely descriptive and inconsistent across individual studies.

Despite these strengths, several limitations should be acknowledged. Most included studies employed cross-sectional designs, limiting the ability to establish temporality or causality between poor glycemic control and impaired pulmonary function. In addition, adjustment for important confounding factors was incomplete across several studies. Pulmonary function is influenced by multiple demographic, anthropometric, lifestyle, nutritional, and disease-related factors, including age, sex, height, body weight, body mass index, race or ethnicity, smoking status, physical activity, diet or nutritional status, muscle mass, and duration of diabetes. Because these variables were inconsistently reported and adjusted for in the primary studies, the pooled estimates may be affected by residual confounding. Therefore, the observed mean differences should be interpreted cautiously and may not fully represent physiological impairment relative to population-specific reference standards.

An additional limitation relates to the temporal interpretation of HbA1c. HbA1c reflects average glycemic exposure over approximately the preceding 8–12 weeks, whereas the structural mechanisms proposed to explain diabetes-associated pulmonary dysfunction, such as extracellular matrix glycation, advanced glycation end-product accumulation, and pulmonary microangiopathy, likely develop over years of chronic metabolic exposure. Therefore, a single baseline HbA1c measurement may not accurately capture cumulative glycemic burden. In cross-sectional studies, this temporal mismatch may lead to exposure misclassification. Individuals with long-standing poor glycemic control who achieved improved HbA1c shortly before spirometry could be categorized as having good control despite established pulmonary structural changes. Conversely, individuals with recent HbA1c deterioration but limited cumulative hyperglycemic exposure could be categorized as having poor control. Such misclassification may dilute between-group differences and bias pooled estimates toward the null.

Because FEV1 and FVC were pooled as absolute values in liters rather than percent-predicted values, z-scores, or lower-limit-of-normal classifications, the pooled mean differences should be interpreted as group-level differences rather than definitive evidence of clinically abnormal pulmonary function. Absolute spirometric values are influenced by age, sex, height, ethnicity, and body size; therefore, residual demographic and anthropometric differences may have influenced the estimates.

## Conclusion

This meta-analysis demonstrates that poor glycemic control in type 2 diabetes mellitus is associated with lower absolute FEV1 and FVC. The preserved or slightly higher FEV1/FVC ratio suggests that the observed reductions in spirometric volumes were not accompanied by a proportional reduction in the ratio, and may be compatible with possible restrictive physiology rather than obstructive impairment. However, true restriction cannot be confirmed without lung volume measurements, particularly total lung capacity. Because the included studies were observational, relied on absolute spirometric values, and were incompletely adjusted for important confounders, the results should be interpreted cautiously as group-level associations. Further longitudinal studies using percent-predicted values, z-scores, lung volume measurements, repeated HbA1c assessment, and comprehensive adjustment for confounding factors are needed.

## Supporting information

S1 Appendix Detailed search strategy and additional search methods. (DOCX)

S2 Appendix PRISMA 2020 checklist. (DOCX)

S3 Appendix Certainty of evidence assessment. (DOCX)

S4 Appendix Funnel plots for publication bias. (DOCX)

S5 Appendix Risk of bias assessment. (DOCX)
