# Supplementary material for: HbA1c and pulmonary function in type 2 diabetes mellitus: A systematic review and meta-analysis
Source: PLoS One. 2026 Aug 13;21(8):e0355628. doi: 10.1371/journal.pone.0355628 (PMC13472463; doi:10.1371/journal.pone.0355628)
Supplement: Supplementary file 1 [file pone.0355628.s001.docx]

# S1 Appendix – Search Methods

## Appendix A. Detailed Search Strategy

# Databases and Search Period

A comprehensive literature search was conducted in **PubMed/MEDLINE, Scopus, and Web of Science Core Collection**. Each database was searched from inception to 25 December 2025. The final search update was performed on 25 December 2025. No restrictions were applied regarding language, publication year, geographic location, or study setting.

*Records retrieved in the final database search before deduplication are shown below and correspond to the PRISMA flow diagram.*

# Search Concepts and Term Selection

The strategy was structured around three concepts: **(1) type 2 diabetes mellitus, (2) glycemic control assessed using HbA1c, and (3) pulmonary function or spirometry**. In PubMed/MEDLINE, Medical Subject Headings (MeSH) were combined with free-text terms searched in the title and abstract fields. Scopus and Web of Science strategies used their corresponding title, abstract, keyword, or topic fields. Synonyms and British/American spelling variants were included, and the concepts were combined using the Boolean operators AND and OR.

# PubMed/MEDLINE

**Fields used:** MeSH and Title/Abstract. The following representative final strategy was used:

("Diabetes Mellitus, Type 2"[Mesh] OR "type 2 diabetes mellitus"[Title/Abstract] OR "type 2 diabetes"[Title/Abstract] OR "type II diabetes"[Title/Abstract] OR T2DM[Title/Abstract])
AND
("Hemoglobin A, Glycosylated"[Mesh] OR HbA1c[Title/Abstract] OR "glycated hemoglobin"[Title/Abstract] OR "glycated haemoglobin"[Title/Abstract] OR "glycosylated hemoglobin"[Title/Abstract] OR "glycosylated haemoglobin"[Title/Abstract] OR "glycemic control"[Title/Abstract] OR "glycaemic control"[Title/Abstract])
AND
("Respiratory Function Tests"[Mesh] OR "Spirometry"[Mesh] OR "pulmonary function"[Title/Abstract] OR "pulmonary function test"[Title/Abstract] OR "pulmonary function tests"[Title/Abstract] OR "lung function"[Title/Abstract] OR spirometry[Title/Abstract] OR "forced expiratory volume"[Title/Abstract] OR FEV1[Title/Abstract] OR "forced vital capacity"[Title/Abstract] OR FVC[Title/Abstract])

# Scopus

**Field used:** TITLE-ABS-KEY. The database syntax was adapted as follows:

TITLE-ABS-KEY("type 2 diabetes mellitus" OR "type 2 diabetes" OR "type II diabetes" OR T2DM)
AND
TITLE-ABS-KEY(HbA1c OR "glycated hemoglobin" OR "glycated haemoglobin" OR "glycosylated hemoglobin" OR "glycosylated haemoglobin" OR "glycemic control" OR "glycaemic control")
AND
TITLE-ABS-KEY("pulmonary function" OR "pulmonary function test*" OR "lung function" OR spirometr* OR "forced expiratory volume" OR FEV1 OR "forced vital capacity" OR FVC)

# Web of Science Core Collection

**Field used:** Topic (TS), which searches titles, abstracts, author keywords, and Keywords Plus. The database syntax was adapted as follows:

TS=("type 2 diabetes mellitus" OR "type 2 diabetes" OR "type II diabetes" OR T2DM)
AND
TS=(HbA1c OR "glycated hemoglobin" OR "glycated haemoglobin" OR "glycosylated hemoglobin" OR "glycosylated haemoglobin" OR "glycemic control" OR "glycaemic control")
AND
TS=("pulmonary function" OR "pulmonary function test*" OR "lung function" OR spirometr* OR "forced expiratory volume" OR FEV1 OR "forced vital capacity" OR FVC)

# Additional Search Methods

- Reference lists of all included studies were screened manually for additional eligible primary studies.
- Reference lists of relevant review articles were screened to identify primary studies not captured by the electronic database searches.

# Search Documentation

Search strings were adapted only to the syntax and field structure of each database while preserving the same three conceptual blocks. Quotation marks indicate phrase searching, and the asterisk (*) indicates truncation in Scopus and Web of Science. The reported search dates and record counts correspond to the final search used for the review and the PRISMA 2020 flow diagram.
