# Supplementary material for: HbA1c and pulmonary function in type 2 diabetes mellitus: A systematic review and meta-analysis
Source: PLoS One. 2026 Aug 13;21(8):e0355628. doi: 10.1371/journal.pone.0355628 (PMC13472463; doi:10.1371/journal.pone.0355628)
Supplement: Supplementary file 3 [file pone.0355628.s003.docx]

**S3. Certainty of Evidence by Outcome (GRADE)**

All included studies were observational and therefore started at low certainty. No outcome was upgraded because there was no prespecified evidence of a large effect, a dose-response gradient, or residual confounding that would be expected to increase the observed association.

| **Outcome** | **No. of studies** | **Study design** | **Risk of bias** | **Inconsistency** | **Indirectness** | **Imprecision** | **Publication bias** | **Overall certainty (GRADE)** |
| --- | --- | --- | --- | --- | --- | --- | --- | --- |
| FEV₁ | 6 | Observational | Not serious overall; some concerns due to residual confounding | Not serious (I² = 0%) | Not serious | Not serious | Undetected; assessment limited | **Low** |
| FVC | 6 | Observational | Not serious overall; some concerns due to residual confounding | Not serious (I² = 0%) | Not serious | Not serious | Undetected; assessment limited | **Low** |
| FEV₁/FVC ratio | 5 | Observational | Not serious overall; some concerns due to residual confounding | Not serious (I² = 0%) | Not serious | Not serious | Undetected; assessment limited | **Low** |

**Risk of bias:** Some included studies incompletely identified or adjusted for important confounders, including age, sex, height, body size or BMI, smoking status, ethnicity, diabetes duration, physical activity, nutritional status, and muscle mass. These concerns were considered in interpretation but were not judged sufficient to warrant an additional full-level downgrade beyond the baseline low certainty of observational evidence.

**Inconsistency:** No important statistical inconsistency was identified for any outcome (I² = 0%).

**Imprecision:** The pooled confidence intervals excluded the null effect and were considered sufficiently precise for the group-level associations evaluated.

**Publication bias:** Formal tests for publication bias were not performed because fewer than 10 studies contributed to each outcome. Funnel-plot inspection was exploratory; publication bias could not be excluded.

**Overall certainty:** The certainty of evidence was rated low for FEV₁, FVC, and the FEV₁/FVC ratio because all evidence came from observational studies and no GRADE upgrading criteria were met.
