# Supplementary material for: HbA1c and pulmonary function in type 2 diabetes mellitus: A systematic review and meta-analysis
Source: PLoS One. 2026 Aug 13;21(8):e0355628. doi: 10.1371/journal.pone.0355628 (PMC13472463; doi:10.1371/journal.pone.0355628)
Supplement: Supplementary file 4 [file pone.0355628.s004.docx]

# **S4 Appendix – Funnel Plot Publication Bias**

**S4.1. FEV1**

S4.1. Funnel plot for the meta-analysis of FEV1, based on six studies. The effect measure was the mean difference in liters. Visual inspection did not show obvious asymmetry; however, the small number of studies limits the reliability of funnel-plot interpretation.

**S4.2. FVC**

S4.2. Funnel plot for the meta-analysis of FVC, based on six studies. The effect measure was the mean difference in liters. Visual inspection did not show obvious asymmetry; however, the small number of studies limits the reliability of funnel-plot interpretation.

**S4.3. FEV1/FVC ratio**

S4.3. Funnel plot for the meta-analysis of the FEV1/FVC ratio, based on five studies. Values were harmonized into percentage points, and the effect measure was the mean difference in percentage points. Visual inspection did not show obvious asymmetry; however, the assessment was exploratory and publication bias could not be excluded.
