# Supplementary material for: HbA1c and pulmonary function in type 2 diabetes mellitus: A systematic review and meta-analysis
Source: PLoS One. 2026 Aug 13;21(8):e0355628. doi: 10.1371/journal.pone.0355628 (PMC13472463; doi:10.1371/journal.pone.0355628)
Supplement: Supplementary file 5 [file pone.0355628.s005.docx]

# **S5 Appendix – Risk of Bias Assessment Using the Original Eight JBI Items**

| **Study (Year)** | **Q1** | **Q2** | **Q3** | **Q4** | **Q5** | **Q6** | **Q7** | **Q8** | **Overall appraisal** |
| --- | --- | --- | --- | --- | --- | --- | --- | --- | --- |
| Anandhalakshmi et al. (2013) | Yes | Unclear | Yes | Yes | No | No | Yes | Yes | Include |
| Maan et al. (2021) | Yes | Yes | Yes | Yes | Yes | Yes | Yes | Yes | Include |
| Singh et al. (2023) | Yes | Unclear | Yes | Yes | No | Unclear | Yes | Yes | Include |
| Senthilnathan et al. (2016) | Yes | Unclear | Yes | Yes | Unclear | No | Yes | Yes | Include |
| Barik et al. (2025) | Yes | Yes | Yes | Yes | Yes | Yes | Yes | Yes | Include |
| Dennis et al. (2010) | Yes | Yes | Yes | Yes | Yes | Yes | Yes | Yes | Include |

**Legend:** Yes = criterion met; No = criterion not met; Unclear = insufficient information; Not applicable = criterion not relevant.

Interpretation: The main methodological concerns were incomplete identification of confounding factors and/or insufficiently described strategies for addressing confounding in Anandhalakshmi et al., Singh et al., and Senthilnathan et al. Maan et al., Barik et al., and Dennis et al. met all eight checklist criteria. All studies were retained because they met the eligibility criteria and provided extractable data; item-level limitations were considered in the certainty-of-evidence assessment and interpretation of pooled estimates.
