# Supplementary material for: HbA1c and pulmonary function in type 2 diabetes mellitus: A systematic review and meta-analysis
Source: PLoS One. 2026 Aug 13;21(8):e0355628. doi: 10.1371/journal.pone.0355628 (PMC13472463; doi:10.1371/journal.pone.0355628)
Supplement: Supplementary file 6 [file pone.0355628.s007.docx]

**Response to Reviewers**

**Manuscript ID:** PONE-D-25-68675
**Title:** ***HbA1c and Pulmonary Function in Type 2 Diabetes Mellitus: A Systematic Review and Meta-Analysis***

**Dear Editor and Reviewer,**

We sincerely thank the Academic Editor and Reviewer for their careful evaluation of our manuscript and for the constructive comments provided. We greatly appreciate the positive assessment of the novelty, clinical relevance, methodological rigor, and consistency of our findings. We have carefully revised the manuscript in response to all comments and believe that the revised version has been substantially improved.

Below, we provide a detailed point-by-point response.

**Responses to the Academic Editor and Journal Requirements**

**1. Please ensure that your manuscript meets PLOS ONE's style requirements, including those for file naming.**

**Response:**
Thank you for this reminder. We have revised the manuscript to better align with PLOS ONE style requirements, including terminology consistency, figure caption presentation, supporting information captions, and overall manuscript organization. We have also reviewed file naming and manuscript structure prior to resubmission.

**2. We note that the grant information you provided in the ‘Funding Information’ and ‘Financial Disclosure’ sections do not match. Please ensure consistency.**

**Response:**
Thank you for identifying this issue. We acknowledge the inconsistency between the funding information entered in the submission system and the wording in the manuscript. We have revised our funding disclosure for consistency and clarity in the resubmitted materials.

Our amended funding statement is as follows:
**“This study received institutional support from Warmadewa University. No specific grant number was assigned. The funders had no role in study design, data collection and analysis, decision to publish, or preparation of the manuscript.”**

If the journal prefers a simplified statement in the submission form, we are also prepared to use:
**“The authors received no specific funding for this work.”**

We respectfully request that the editorial office update the submission record accordingly.

**3. Please clarify the sources of funding and the role of the funders.**

**Response:**
Thank you. We have clarified the funding source and the role of the funders. Specifically, we now state that the study received institutional support from Warmadewa University, that no specific grant number was assigned, and that the funders had no role in study design, data collection and analysis, decision to publish, or preparation of the manuscript.

**4. Please remove any funding-related text from the manuscript outside the Funding Statement.**

**Response:**
Thank you for this clarification. We have revised the manuscript accordingly and removed funding-related wording from sections where it should not appear as part of the published manuscript narrative. Funding information will be presented only in the appropriate funding declaration during resubmission.

**5. Please include a separate caption for each figure in your manuscript.**

**Response:**
Thank you. We have revised the manuscript to include a separate caption for each figure in the main manuscript file. Figure captions are now clearly presented for Figure 1 through Figure 4.

**6. We note you have included a table to which you do not refer in the text of your manuscript. Please ensure that you refer to Table 1 in your text.**

**Response:**
Thank you. We have revised the Results section to explicitly refer to Table 1 in the text. The characteristics of the included studies are now directly referenced in the narrative.

**7. Please include captions for your Supporting Information files at the end of your manuscript, and update any in-text citations to match accordingly.**

**Response:**
Thank you. We have added the Supporting Information section at the end of the manuscript and included captions for all supplementary files. We also revised the in-text citations to improve consistency with the supplementary materials.

**8. Please review your reference list to ensure that it is complete and correct. If you have cited papers that have been retracted, please include the rationale for doing so in the manuscript text, or remove these references and replace them with relevant current references.**

**Response:**
Thank you. We carefully reviewed the reference list for completeness and accuracy. We corrected several bibliographic inaccuracies, including author names, journal titles, publication years, volume/issue numbers, and page ranges where needed. We did not identify any retracted references among the studies cited in the revised manuscript. All relevant corrections have been incorporated into the revised reference list.

**Responses to Reviewer #1**

We sincerely thank Reviewer #1 for the thoughtful and encouraging review. We greatly appreciate the positive comments regarding the originality, methodological rigor, consistency of findings, and clinical relevance of our study.

**Comment 1. Search Strategy and Timeframe**

*The stated search period (“June 2025 to December 2025”) is unconventional and potentially confusing. Clarification is required: Was this the date range of the search or the publication years searched? If older studies (e.g., Dennis et al. 2010) were included, the search timeframe should reflect that accurately. Consider adding the complete search strings (not only in supplementary material but summarized briefly in the main text).*

**Response:**
We thank the Reviewer for this important observation. We agree that the wording in the original manuscript may have been confusing. We have revised the manuscript to clarify that the databases were searched **from database inception to 25 December 2025**, while the actual search process was conducted between **June 2025 and 25 December 2025**. We also clarified that no publication-year restriction was applied. In addition, we retained the full electronic search strategies in the supplementary file and summarized the main search concepts and key terms in the Methods section.

**Comment 2. Confounding and Adjustment**

*Several included studies did not adequately adjust for age, height, sex, smoking status, BMI, or diabetes duration. The reliance on absolute spirometric values further amplifies this concern. Although justified, this trade-off should be more explicitly framed as a methodological decision with consequences for interpretation.*

**Response:**
We thank the Reviewer for this insightful comment. We agree that residual confounding is an important limitation, particularly because several included studies did not adequately adjust for key determinants of pulmonary function, including age, sex, height, smoking status, body mass index, and duration of diabetes.

In response, we strengthened the Limitations section to more explicitly frame the use of absolute spirometric values as a methodological decision with important interpretive consequences. We now clarify that absolute spirometric values were synthesized because they were the most consistently reported measures across studies and allowed direct cross-study comparability. However, we also explicitly acknowledge that raw lung volumes are influenced by demographic and anthropometric characteristics and therefore should be interpreted cautiously.

**Comment 3. Interpretation of FEV₁/FVC Findings**

*The reduction in FEV₁/FVC ratio, while statistically significant, is modest. Traditionally, restrictive lung disease is associated with a preserved or increased FEV₁/FVC ratio. The manuscript would benefit from a clearer discussion on whether this reflects mixed ventilatory physiology, small airway involvement, measurement variability, or early airflow limitation related to inflammation or autonomic dysfunction.*

**Response:**
We thank the Reviewer for this important point. We agree that the observed reduction in the FEV₁/FVC ratio requires a more nuanced interpretation. In the revised manuscript, we expanded the discussion of this finding and clarified that the modest reduction in FEV₁/FVC should not be interpreted as evidence of pure restrictive physiology alone.

We now state that classical restrictive ventilatory impairment is typically associated with a preserved or increased FEV₁/FVC ratio, and that the lower ratio observed in our analysis may instead reflect a more complex physiological pattern. We further discuss several possible explanations, including mixed ventilatory physiology, early small-airway involvement, subtle airflow limitation related to chronic inflammation or autonomic dysfunction, and inter-study variability in spirometric measurement and reporting.

**Comment 4. Limited Study Number and Geographic Concentration**

*Only six studies were included, with predominant representation from South Asia. Generalizability to other populations may be limited. This should be more clearly acknowledged in the limitations.*

**Response:**
We thank the Reviewer for highlighting this important issue. We agree that the limited number of included studies and the geographic concentration of most studies in South Asia may limit the generalizability of our findings. Accordingly, we revised the Limitations section to explicitly acknowledge this issue and to note that further studies from more diverse global settings, including African, European, and North American populations, are needed to confirm the consistency and generalizability of these associations.

**Comment 5. Language and Style**

*Minor grammatical and typographical issues are present (e.g., “by, quantifying” in the Introduction). Occasional sentence length could be reduced for clarity.*

**Response:**
Thank you. We carefully proofread the manuscript and revised grammatical, typographical, and stylistic issues throughout the text. This included correction of typographical errors, revision of awkward or overly long sentences for clarity, and overall improvement of readability across the Abstract, Introduction, Methods, Results, Discussion, and end-matter sections.

**Comment 6. Terminology**

*Use consistent terminology throughout (e.g., “crosssectional” → “cross-sectional”). Standardize HbA1c cutoff descriptions to avoid confusion.*

**Response:**
Thank you for this helpful suggestion. We revised the manuscript to ensure more consistent terminology throughout. In particular, terms such as “crosssectional” were corrected to “cross-sectional,” and descriptions of HbA1c cutoffs were standardized where possible. We also clarified that glycemic control thresholds varied across studies and were therefore reported according to each study’s original definitions.

**Additional Revisions Made by the Authors**

In addition to the points raised by the Reviewer and Editor, we made several further improvements to strengthen the manuscript. These include revision of the data availability statement to align with PLOS ONE policy, addition of supporting information captions, correction of bibliographic inaccuracies in the reference list, and refinement of the Results and Discussion sections for clarity and internal consistency.

We hope that the revised manuscript adequately addresses all concerns raised by the Editor and Reviewer. We are grateful for the opportunity to revise our work and sincerely appreciate the thoughtful comments, which have helped us improve the manuscript substantially.

Sincerely,
**Kadek Surya Atmaja**
on behalf of all authors
