# Supplementary material for: HbA1c and pulmonary function in type 2 diabetes mellitus: A systematic review and meta-analysis
Source: PLoS One. 2026 Aug 13;21(8):e0355628. doi: 10.1371/journal.pone.0355628 (PMC13472463; doi:10.1371/journal.pone.0355628)
Supplement: Supplementary file 7 [file pone.0355628.s008.docx]

**Reviewer Assessment**

*HbA1c and Pulmonary Function in Type 2 Diabetes Mellitus: A Systematic Review and Meta-Analysis*

| **Journal / Platform** | PLOS ONE |
| --- | --- |
| **Manuscript number** | PONE-D-25-68675R2 |
| **Article type** | Research Article |
| **Overall recommendation** | Major revision before acceptance |

# **Overall assessment**

This systematic review and meta-analysis evaluate the association between poor glycemic control, measured by HbA1c, and pulmonary function in adults with type 2 diabetes mellitus. The topic is appropriate and clinically meaningful. The overall direction of findings is consistent, with lower FEV1 and FVC among individuals with poorer glycemic control. Nevertheless, the manuscript still contains internal inconsistencies and methodological overstatements that must be addressed.

# **Major comments**

## 1. PRISMA flow numbers are internally inconsistent

**Reviewer concern:** The study selection section reports 1,243 records identified, 948 records screened after duplicate removal, 901 records excluded at title/abstract screening, 44 full-text articles assessed, 41 full-text articles excluded, and 6 studies included. These numbers do not align. If 948 records were screened and 901 were excluded, then 47 full-text articles should have been assessed, not 44. If 44 full-text articles were assessed and 41 were excluded, then only 3 studies should remain, not 6. This is a major reporting problem and must be corrected in both the text and the PRISMA figure.

**Required revision:** Please revise the PRISMA flow diagram and study selection text so that all numbers are internally consistent.

## 2. Inconsistency in the number of studies included for FEV1/FVC ratio

**Reviewer concern:** The Study Characteristics section states that four studies provided FEV1/FVC data, but the Results section states that five studies contributed data to the FEV1/FVC ratio meta-analysis. The table appears to show FEV1/FVC data for Anandhalakshmi, Maan, Singh, and Senthilnathan only, whereas Barik and Dennis are listed as providing only FEV1 and FVC. This discrepancy directly affects the validity of the FEV1/FVC pooled estimate.

**Required revision:** Please clearly state the exact number of studies and participants contributing to each pooled outcome: FEV1, FVC, and FEV1/FVC.

## 3. GRADE rating of “moderate certainty” may be overstated

**Reviewer concern:** The manuscript states that all included studies were observational, so the initial certainty of evidence was low, but later concludes that the certainty of evidence was moderate for all outcomes. This needs stronger justification. Several included studies had incomplete adjustment for key confounders such as age, sex, BMI, smoking status, height, ethnicity, diabetes duration, physical activity, nutritional status, and muscle mass. The manuscript itself acknowledges residual confounding as a major limitation.

**Required revision:** Please either downgrade the certainty to low or provide a clear GRADE justification for upgrading from low to moderate, such as large effect size, dose-response gradient, or residual confounding that would likely reduce rather than increase the observed association.

## 4. Use of absolute spirometric values limits interpretability

**Reviewer concern:** The manuscript includes only absolute spirometric values in liters. This improves direct pooling but limits clinical interpretation because FEV1 and FVC are strongly influenced by age, sex, height, ethnicity, and body size. This limitation is mentioned but should be emphasized more strongly because it directly affects cross-study comparability and interpretation of clinical significance.

**Required revision:** Please avoid implying that the observed absolute reductions necessarily represent clinically meaningful impairment without percent-predicted values, lower limit of normal, or total lung capacity.

## 5. The FEV1/FVC finding conflicts with a purely restrictive interpretation

**Reviewer concern:** The manuscript suggests a restrictive pattern because both FEV1 and FVC are reduced. However, the pooled FEV1/FVC ratio is also reported as reduced. Classical restrictive ventilatory impairment usually has a preserved or increased FEV1/FVC ratio. Although the authors acknowledge this later, the abstract and conclusion still present the findings too strongly as a restrictive lung pattern.

**Required revision:** Please soften “restrictive lung pattern” throughout the manuscript. Prefer phrases such as “reduced spirometric volumes suggestive of possible restrictive physiology” or “reduced lung volumes without definitive confirmation of restriction.” True restriction requires lung volume measurement, particularly total lung capacity.

## 6. Modification of the JBI risk-of-bias tool requires stronger justification

**Reviewer concern:** The manuscript states that the original JBI checklist has eight items, but two items were combined because they were conceptually overlapping, resulting in seven domains. Modifying a validated tool can be questioned unless the process is transparent.

**Required revision:** Please specify exactly which two JBI items were combined and provide both the original eight-item assessment and the modified seven-domain reconciliation in the supplement.

## 7. Search strategy description is incomplete in the main manuscript

**Reviewer concern:** The manuscript describes the search terms broadly and refers readers to the supplement for the detailed strategy. For reproducibility, the main Methods section should briefly clarify whether MeSH terms, title/abstract terms, synonyms, and spelling variants were used.

**Required revision:** Please include at least one representative search string in the manuscript or ensure the supplementary file provides complete database-specific Boolean strategies.

## 8. PROSPERO timing requires clarification

**Reviewer concern:** The manuscript states that the protocol was prospectively registered in PROSPERO, but it does not clearly state the date of registration or whether registration occurred before screening and data extraction were completed.

**Required revision:** Please add the date of PROSPERO registration and clarify whether registration occurred before completion of study selection and data extraction. If registration occurred after screening began, the wording “prospectively registered” should be modified.

# Suggested exact wording for key revisions

## For absolute spirometric values

**Suggested wording:**

Because spirometric values were pooled as absolute volumes rather than percent-predicted values, the observed mean differences should be interpreted as group-level associations rather than definitive evidence of clinically abnormal lung function.

## For clinical implication

**Suggested wording:**

These findings support awareness of possible pulmonary involvement in patients with poorly controlled or long-standing type 2 diabetes, particularly when respiratory symptoms or additional cardiometabolic risk factors are present.

## For restrictive interpretation

**Suggested wording:**

The reductions in FEV1 and FVC suggest lower spirometric volumes among individuals with poor glycemic control; however, confirmation of true restrictive ventilatory impairment requires lung volume assessment, particularly total lung capacity.

# Minor and editorial comments

1. **Abstract Methods sentence is grammatically incomplete.** Revise to: “We searched PubMed, Scopus, and Web of Science from database inception to 25 December 2025. Studies were eligible if they compared spirometric outcomes between adults with type 2 diabetes categorized by good versus poor glycemic control based on HbA1c.”
2. **Duplicate sentences are present.** Remove repeated sentences in the Introduction, Data Extraction, and Study Characteristics sections.
3. **Section heading should be corrected.** Change “Result” to “Results.”
4. **Capitalization and figure citation need correction.** Use “PRISMA” consistently and write “Figure 1” rather than “fig 1.”
5. **Spelling errors remain.** Correct “metanalysis” to “meta-analysis,” “Data Availabilitiy” to “Data Availability,” and “alveolar-capirally” to “alveolar-capillary.”
6. **Extra punctuation remains.** Remove double periods such as “duration..”, “study..”, and “reporting of this study..”.
7. **Reference list formatting is problematic.** References 6–9 appear disrupted, with numbering and author details broken across lines. Recheck the full reference list against journal style and original source metadata.
8. **ATS/ERS claim requires verification.** State this only if each included study explicitly reported ATS/ERS-compliant spirometry. Otherwise soften to “Most included studies reported standardized spirometric assessment; where available, spirometry was performed according to ATS/ERS-based procedures.”

# Outcome-specific clarification table requested from authors

| **Outcome** | **Number of studies** | **Total participants** |
| --- | --- | --- |
| FEV1 | 6 | To be stated |
| FVC | 6 | To be stated |
| FEV1/FVC ratio | Clarify: 4 or 5 | To be stated |

# Final reviewer recommendation

**Recommendation: Major revision**
