# Supplementary material for: HbA1c and pulmonary function in type 2 diabetes mellitus: A systematic review and meta-analysis
Source: PLoS One. 2026 Aug 13;21(8):e0355628. doi: 10.1371/journal.pone.0355628 (PMC13472463; doi:10.1371/journal.pone.0355628)
Supplement: Supplementary file 8 [file pone.0355628.s009.docx]

**Revision: Temporal limitation of the Biomarker (HbA1c)**

The authors cited a long-term structural mechanism, such as extracellular matrix glycation and pulmonary microangiopathy to justify why the lung function drops. However, while the structural tissue remodeling takes years of chronic hyperglycemia to develop, HBA1c is a short-term biomarker reflecting glycaemic status over only the preceding 8-12 weeks.

The authors are requested to address this temporal mismatch:

1. Please add a brief discussion to the limitation section noting that relying on a short-term baseline biomarker to explain chronic, structural organ damage is a key limitation, especially given the cross-sectional nature of the underlying date.
2. Please comment on how potential patient misclassification might affect your pooled mean differences. For example, if a patient with uncontrolled diabetes (including lung changes) for ten years, who achieved strict glycemic control only in the 3 months preceding the study would be misclassified into the “good control” cohort, potentially blurring or underestimating the true impact of chronic hyperglycemia.
