# Supplementary material for: HbA1c and pulmonary function in type 2 diabetes mellitus: A systematic review and meta-analysis
Source: PLoS One. 2026 Aug 13;21(8):e0355628. doi: 10.1371/journal.pone.0355628 (PMC13472463; doi:10.1371/journal.pone.0355628)
Supplement: Supplementary file 9 [file pone.0355628.s010.docx]

**Response to Reviewers**

**Dear Editor and Reviewers,**

We sincerely thank the Academic Editor and Reviewer #2 for the careful evaluation of our revised manuscript and for the constructive comments that have helped us further improve the clarity, rigor, and interpretability of the manuscript. We are grateful that Reviewer #2 indicated that all previous comments had been addressed, that the statistical analysis was considered appropriate and rigorous, that the data availability was acceptable, and that the manuscript was written in standard English. In this revision, we have addressed the remaining concerns by strengthening the discussion of residual confounding, clarifying the limitations of using absolute spirometric values, tempering causal language throughout the manuscript, correcting reference no. 7, rechecking the full reference list, and correcting the bibliographic details of reference no. 20 based on the original article.

For ease of review, we provide below a concise summary table followed by a detailed point-by-point response. Reviewer comments are reproduced in italics, followed by our responses and the specific changes made to the manuscript.

# Summary Table of Revisions

| **No.** | **Issue addressed** | **Concern** | **Revision made** | **Location in manuscript** |
| --- | --- | --- | --- | --- |
| 1 | Residual confounding and generalizability | Reviewer noted that factors such as weight, BMI, race, diet, and muscle mass were not sufficiently considered. | Expanded the manuscript to explicitly acknowledge residual confounding from demographic, anthropometric, lifestyle, nutritional, and disease-related variables, including age, sex, height, body weight, BMI, race/ethnicity, smoking status, physical activity, diet/nutritional status, muscle mass, and diabetes duration. | Introduction; Data Extraction; Risk of Bias Assessment; Discussion; Strengths and Limitations; Conclusion |
| 2 | Use of absolute spirometric values | Absolute FEV1 and FVC values may be influenced by body size, height, sex, ethnicity, and muscle mass. | Added clarification that pooling absolute values in liters may increase susceptibility to confounding and may not fully represent physiological impairment relative to population-specific percent-predicted reference standards. | Discussion; Strengths and Limitations |
| 3 | Causal interpretation | Because included studies were observational, causal conclusions should be avoided. | Replaced stronger causal phrasing with more cautious language, including “associated with,” “may contribute to,” “should be interpreted cautiously,” and “hypothesis-generating.” | Discussion; Clinical interpretation; Conclusion |
| 4 | Reference no. 7 | Reviewer requested that reference no. 7 be rechecked for validity and citation accuracy. | Corrected the mismatched reference and aligned the relevant text with the corrected citation on systemic inflammation/CRP and reduced lung function. | Introduction/Discussion; References |
| 5 | Reference list accuracy | PLOS ONE requested that the reference list be reviewed for completeness and correctness. | Rechecked references for author names, titles, journal names, volume/issue numbers, page ranges, and DOI details where available. No evidence of retraction was identified among verified references. | References |
| 6 | Funding/disclosure consistency | Submission materials should use consistent financial disclosure wording. | Reviewed funding and competing interest statements for consistency and retained the statement that the authors received no specific funding and have no competing interests. | Financial disclosure; Conflict of Interest |
| 7 | Figures and supporting information | PLOS requested that figure requirements and files be checked. | Reviewed figure captions, PRISMA flow diagram, forest plots, and supporting information labels for consistency with the manuscript text. | Figure captions; Supporting Information |

# Files Submitted with the Revision

- Response to Reviewers: this document.
- Revised Manuscript with Track Changes: a marked-up version highlighting all changes made in response to the Academic Editor and Reviewer #2.
- Manuscript: a clean, unmarked version of the revised manuscript.
- Updated figure and supporting information files, as applicable.

# Response to the Academic Editor and Journal Requirements

## Journal requirement: Submit a letter responding to each point raised by the Academic Editor and reviewer(s).

**Response:** We have prepared this detailed point-by-point response letter and uploaded it as a separate file labeled “Response to Reviewers.”

## Journal requirement: Submit a marked-up copy of the revised manuscript and a clean unmarked version.

**Response:** We have prepared both a tracked/marked-up version labeled “Revised Manuscript with Track Changes” and a clean version labeled “Manuscript.”

## Journal requirement: Review the reference list to ensure that it is complete and correct.

**Response:** We carefully rechecked the reference list for bibliographic accuracy and consistency. Several entries were corrected for author names, article titles, journal titles, volume/issue details, page ranges, and DOI information where applicable. In particular, we corrected reference no. 7, as specifically requested by Reviewer #2, and corrected reference no. 20 after verifying the original article. We also reviewed the reference list for potentially retracted articles and found no evidence among the verified references that any cited work had been retracted.

## Journal note: If changes to financial disclosure are needed, include the updated statement in the cover letter.

**Response:** We reviewed the funding and disclosure statements for consistency. The revised manuscript uses a consistent statement indicating that the authors received no specific funding for this work, unless otherwise required by the submission system. The competing interest statement remains unchanged: the authors declare no competing interests.

## Journal note: Review figure requirements and resubmit figure files as needed.

**Response:** We reviewed the figure captions and figure files for clarity and consistency. We ensured that the PRISMA flow diagram, forest plots, and supplementary figure materials are consistently labeled and correspond to the text.

## Journal note: Protocols.io recommendation.

**Response:** This study is a systematic review and meta-analysis rather than a laboratory protocol study. The review was prospectively registered in PROSPERO, and detailed search strategies are provided in the Supporting Information. Therefore, a protocols.io laboratory protocol deposit is not applicable to this manuscript.

# Response to Reviewer #2

## General assessment by Reviewer #2

**Reviewer comment/assessment:** All comments have been addressed.

**Response:** We thank the reviewer for acknowledging that the comments raised in the previous round have been addressed. In the present revision, we focused on the remaining concerns regarding residual confounding, generalizability, and reference accuracy.

## Technical soundness and support for conclusions

**Reviewer comment/assessment:** Partly.

**Response:** We understand that this assessment relates primarily to the reviewer’s concern that several potential confounding factors were not sufficiently considered. We agree that the observational nature of the included studies and the incomplete adjustment for key determinants of pulmonary function limit the strength of causal interpretation. Accordingly, we revised the manuscript to more clearly state that the pooled results should be interpreted as associations rather than fully adjusted causal effects. We also added detailed discussion of residual confounding in the Methods, Risk of Bias Assessment, Discussion, Strengths and Limitations, and Conclusion sections.

**Specific changes made:**

- Causal language was tempered throughout the manuscript.
- Residual confounding was explicitly discussed as a key limitation.
- The conclusion was revised to emphasize cautious interpretation and the need for future longitudinal studies.

## Statistical analysis

**Reviewer comment/assessment:** Yes.

**Response:** We thank the reviewer for considering the statistical analysis appropriate and rigorous. The primary meta-analytic approach was retained. However, we expanded the limitations to clarify that adjusted pooled analyses, subgroup analyses, sensitivity analyses, and meta-regression could not be performed because the number of eligible studies was limited and because key confounders were inconsistently reported across the primary studies.

**Specific changes made:**

- The limitations now explain why subgroup analysis, meta-regression, and sensitivity analysis by BMI, ethnicity, smoking status, diabetes duration, nutritional status, or muscle mass could not be performed.

## Data availability

**Reviewer comment/assessment:** Yes.

**Response:** We thank the reviewer for confirming that the data underlying the manuscript are fully available. The Data Availability Statement remains: “All relevant data are within the manuscript and its Supporting Information files.”

## Language and presentation

**Reviewer comment/assessment:** Yes.

**Response:** We thank the reviewer for this assessment. We nevertheless re-read the manuscript and made minor language and terminology edits to improve clarity, consistency, and precision. In particular, we replaced language that could imply causality with more cautious wording, such as “associated with,” “may contribute to,” and “should be interpreted cautiously.”

## Main Comment 1: Residual Confounding and Generalizability

**Reviewer comment:** The manuscript highlights an important, yet infrequently discussed and even more poorly understood aspect of diabetes-lung function and glycaemic control. Although the statistical methods and study selection criteria are robustly described, the fact that potential confounding factors such as weight, BMI, race, diet, muscle mass etc have not been considered limit the validity and generalizability of the results; having the same acknowledged as a limitation of the study should suffice in this case.

**Response:** We sincerely thank the reviewer for this constructive and important comment. We agree that residual confounding is an important limitation of this systematic review and meta-analysis. Pulmonary function is strongly influenced by demographic, anthropometric, lifestyle, nutritional, and disease-related factors, including age, sex, height, body weight, body mass index, race or ethnicity, smoking status, physical activity, diet or nutritional status, muscle mass, and duration of diabetes. Because these variables were not uniformly reported or adjusted for across the included primary studies, their influence could not be fully addressed in the pooled analysis.

In response, we revised several sections of the manuscript to explicitly acknowledge this limitation. First, in the Data Extraction section, we clarified that these potential confounders were extracted when available, but were inconsistently reported and therefore could not be uniformly incorporated into quantitative synthesis. Second, in the Risk of Bias Assessment section, we specified that particular attention was given to whether primary studies identified and addressed these confounders. Third, in the Discussion and Strengths and Limitations sections, we explicitly stated that residual confounding may have influenced the magnitude of the association between poor glycemic control and lower spirometric values. Fourth, we revised the Conclusion to avoid overstatement and to emphasize the need for longitudinal studies with standardized spirometric reference values and comprehensive multivariable adjustment.

We also clarified that the use of absolute spirometric values in liters, rather than percent-predicted values, increases susceptibility to confounding by body size, sex, height, ethnicity, and muscle mass. Although absolute FEV1 and FVC values were selected because they were the most consistently reported outcomes across eligible studies, we now explicitly state that these raw values may not fully reflect physiological impairment relative to population-specific reference standards.

**Specific manuscript changes made in response to this comment:**

- Introduction: Expanded the list of potential confounders to include age, sex, height, body weight, BMI, race/ethnicity, smoking status, physical activity, diet/nutritional status, muscle mass, and diabetes duration.
- Data Extraction: Added a statement explaining that these variables were extracted when available but could not be uniformly included in quantitative synthesis because reporting was inconsistent across studies.
- Risk of Bias Assessment: Added a statement that confounding-related domains were judged with attention to whether these factors were identified and addressed.
- Discussion: Added a paragraph emphasizing that the findings should not be interpreted as fully adjusted causal effects and may be influenced by residual confounding.
- Clinical interpretation: Replaced stronger causal statements with more cautious language and framed clinical implications as hypothesis-generating rather than definitive evidence for routine spirometric screening in all patients with type 2 diabetes.
- Strengths and Limitations: Expanded the limitations to explicitly include weight, BMI, race/ethnicity, diet/nutritional status, muscle mass, physical activity, smoking status, and diabetes duration; also clarified the limitations of using absolute spirometric values.
- Conclusion: Revised to state that the findings show an association and should be interpreted cautiously because of observational designs and incomplete adjustment for important confounders.

## Main Comment 2: Reference No. 7

**Reviewer comment:** Attention is drawn to reference no. 7. The authors may want to recheck if this is a valid reference or has been cited correctly.

**Response:** We thank the reviewer for identifying this issue. We rechecked reference no. 7 and found that the previous bibliographic details were mismatched. Specifically, the author list and article details did not consistently correspond to the cited publication. We corrected the citation and revised the corresponding text so that the cited reference now accurately supports the statement regarding low-grade systemic inflammation, C-reactive protein, and reduced lung function.

The corrected reference no. 7 is: Rasmussen F, Mikkelsen D, Hancox RJ, Lambrechtsen J, Nybo M, Hansen HS, Siersted HC. High-sensitive C-reactive protein is associated with reduced lung function in young adults. Eur Respir J. 2009;33(2):382–388. doi:10.1183/09031936.00040708.

We also revised the relevant sentence in the manuscript from a broad statement regarding inflammatory markers to a more precise statement focused on C-reactive protein and low-grade systemic inflammation.

## Additional Correction: Reference No. 20

**Response:** we also rechecked the bibliographic details of reference no. 20 while reviewing the full reference list. We verified the original article and corrected the citation. The previously listed reference incorrectly cited the journal and bibliographic details.

The corrected reference is: Senthilnathan NK, Muthumani L, Nehru D. Effect of glycaemic control (HbA1c) on pulmonary function tests (spirometry) in patients with type 2 diabetes mellitus. J Evid Based Med Healthc. 2016;3(27):1230–1236. doi:10.18410/jebmh/2016/283.

## Additional Correction: Full Reference List Review

**Response:** In addition to the specific correction of reference no. 7, we reviewed the reference list for completeness, accuracy, and consistency. The following types of corrections were made where applicable:

- Author initials and ordering were checked and corrected where necessary.
- Article titles, journal names, volume/issue numbers, page ranges, and DOI information were corrected where necessary.
- References to methodological tools and reporting frameworks, including Rayyan and GRADE, were reviewed and standardized.
- The citations of included primary studies were checked against the original sources where available.
- No evidence of retraction was identified among the verified cited references.

# Closing Statement

We again thank the Academic Editor and Reviewer #2 for their careful assessment and constructive guidance. We believe the revised manuscript now provides a more cautious and methodologically transparent interpretation of the evidence, explicitly acknowledges the limitations related to residual confounding and generalizability, and contains a corrected and more accurate reference list. We hope that the revised manuscript is now suitable for further consideration by PLOS ONE.

Sincerely,

**Kadek Surya Atmaja, MD, Pulmonology Specialist**

On behalf of all authors
