# Supplementary material for: HbA1c and pulmonary function in type 2 diabetes mellitus: A systematic review and meta-analysis
Source: PLoS One. 2026 Aug 13;21(8):e0355628. doi: 10.1371/journal.pone.0355628 (PMC13472463; doi:10.1371/journal.pone.0355628)
Supplement: Supplementary file 10 [file pone.0355628.s011.docx]

Response to Reviewers
Manuscript ID: PONE-D-25-68675
**Title: *HbA1c and Pulmonary Function in Type 2 Diabetes Mellitus: A Systematic Review and Meta-Analysis***

**Dear Editor and Reviewer,**

We sincerely thank the reviewer for the careful and constructive evaluation of our manuscript. The comments were highly valuable and helped us improve the internal consistency, methodological transparency, statistical analysis, and clinical interpretation of the review.

In response, we rechecked the study-selection records, re-extracted the outcome data directly from the six primary studies, reran all meta-analyses using Stata, revised the certainty-of-evidence assessment, returned to the original unmodified eight-item JBI checklist, expanded the search-strategy description, clarified the PROSPERO registration date, and revised the interpretation of the spirometric findings throughout the manuscript.

Our point-by-point responses are provided below.

**Major Comment 1. PRISMA flow numbers are internally inconsistent**

**Reviewer concern:**
The numbers reported for records screened, full-text reports assessed, full-text exclusions, and included studies were internally inconsistent.

**Response:**
We agree with the reviewer. We rechecked the study-selection records and revised both the PRISMA 2020 flow diagram and the Study Selection section so that all numbers are internally consistent.

The corrected study-selection flow is:

- Records identified: 1,243
- Duplicate records removed: 295
- Records screened: 948
- Records excluded at title/abstract screening: 901
- Reports sought for retrieval: 47
- Reports not retrieved: 3
- Full-text reports assessed for eligibility: 44
- Full-text reports excluded: 38
- Studies included in the quantitative synthesis: 6

The 38 full-text exclusions comprised incomplete reporting of spirometric data (n = 12), absence of glycemic-control categories (n = 15), and inappropriate study design (n = 11). Thus, 44 minus 38 correctly results in six included studies.

**Changes made:**
The Study Selection section and Figure 1 have been revised using the corrected PRISMA numbers.

**Major Comment 2. Inconsistency in the number of studies included for the FEV1/FVC ratio**

**Reviewer concern:**
The manuscript inconsistently reported four or five studies contributing to the FEV1/FVC meta-analysis, and the study-characteristics table did not clearly identify all contributing studies.

**Response:**
We agree and have corrected this discrepancy. We re-extracted outcome-specific data directly from the six primary studies and clearly stated the exact number of studies and participants contributing to each pooled outcome:

- FEV1: 6 studies, 944 participants
- FVC: 6 studies, 944 participants
- FEV1/FVC ratio: 5 studies, 801 participants

The five studies contributing to the FEV1/FVC analysis were Anandhalakshmi et al., Maan et al., Singh et al., Senthilnathan et al., and Dennis et al. Barik et al. was not included in the FEV1/FVC meta-analysis because extractable group-specific mean and standard deviation values for the ratio were not available.

Table 1 was revised to show that Dennis et al. contributed FEV1, FVC, and FEV1/FVC data. Table 2 was added to explicitly present the number of studies, participants, and study names contributing to each pooled outcome.

To fully resolve the inconsistency, we re-extracted all spirometric data directly from the six primary studies rather than relying on the previously compiled manuscript dataset. Several studies reported more than two HbA1c categories. For these studies, categories above the study-specific good-control threshold were combined into a single poor-control group using standard formulas for pooled means and pooled standard deviations.

All meta-analyses were then rerun in Stata 17. Stata was selected because it enabled a transparent and reproducible workflow for:

1. Combining multiple HbA1c categories before meta-analysis;
2. Verifying study-specific sample sizes, means, and standard deviations;
3. Applying a uniform effect direction defined as poor glycemic control minus good glycemic control;
4. Harmonizing FEV1/FVC values into percentage points;
5. Generating outcome-specific forest plots and funnel plots; and
6. Maintaining a reproducible analytical dataset and command syntax.

FEV1 and FVC were pooled as mean differences in liters. Because the FEV1/FVC ratio represents the same clinical measure across studies and could be harmonized into percentage points, it was pooled using a mean difference rather than a standardized mean difference. This preserves clinical interpretability.

The revised pooled results were:

- FEV1: MD = −0.16 L, 95% CI −0.25 to −0.07; p < 0.001; I² = 0%
- FVC: MD = −0.24 L, 95% CI −0.35 to −0.14; p < 0.001; I² = 0%
- FEV1/FVC ratio: MD = 1.61 percentage points, 95% CI 0.50 to 2.73; p = 0.005; I² = 0%

The numerical changes were primarily caused by corrected study-level data extraction, consistent outcome coding, category harmonization, and use of the clinically appropriate effect measure.

**Changes made:**

The Study Characteristics section, Tables 1 and 2, Statistical Analysis section, Results, Abstract, Discussion, forest plots, funnel plots, and figure captions have been revised accordingly.

**Major Comment 3. GRADE rating of “moderate certainty” may be overstated**

**Reviewer concern:**
All included studies were observational, and there was insufficient justification for upgrading the certainty of evidence from low to moderate.

**Response:**
We agree. The GRADE assessment has been revised, and the certainty of evidence for FEV1, FVC, and FEV1/FVC is now rated as low.

All outcomes started at low certainty because the included studies were observational. No outcome was upgraded because there was no prespecified evidence of a large effect, a dose-response gradient, or residual confounding that would be expected to increase the observed association.

Although statistical heterogeneity was negligible and the pooled confidence intervals excluded the null effect, several studies incompletely identified or adjusted for important confounders, including age, sex, height, body size or BMI, smoking status, ethnicity, diabetes duration, physical activity, nutritional status, and muscle mass.

**Changes made:**
The Certainty of Evidence Assessment section and Supplementary Appendix S3 have been revised. All three outcomes are now classified as low-certainty evidence.

**Major Comment 4. Use of absolute spirometric values limits interpretability**

**Reviewer concern:**

Absolute FEV1 and FVC values are influenced by age, sex, height, ethnicity, and body size and therefore cannot be interpreted as definitive evidence of clinically abnormal lung function.

**Response:**
We agree and have strengthened this limitation throughout the manuscript. FEV1 and FVC were pooled as absolute volumes in liters because group-specific percent-predicted values, z-scores, or lower-limit-of-normal classifications were not consistently available across the included studies.

We now explicitly state that the pooled mean differences represent group-level associations rather than definitive evidence of clinically abnormal pulmonary function. We also clarify that residual demographic and anthropometric differences may have influenced the estimates.

We avoided interpreting the observed reductions as necessarily clinically meaningful impairment and emphasized the need for future studies to report percent-predicted values, z-scores, lower-limit-of-normal classifications, and lung-volume measurements.

**Changes made:**

The Abstract, Results, Discussion, Strengths and Limitations, and Conclusion have been revised to emphasize the limitations of absolute spirometric values.

**Major Comment 5. The FEV1/FVC finding conflicts with a purely restrictive interpretation**

**Reviewer** concern:
A restrictive ventilatory impairment generally has a preserved or increased FEV1/FVC ratio, whereas the previous manuscript reported a reduced pooled ratio and nevertheless interpreted the findings as restrictive.

**Response:**
We agree. Following direct re-extraction and reanalysis of the primary-study data, the FEV1/FVC ratio was not reduced. The revised analysis showed a small but statistically significant increase in the ratio among participants with poor glycemic control:

MD = 1.61 percentage points, 95% CI 0.50 to 2.73; p = 0.005.

The Abstract, Results, Discussion, and Conclusion have therefore been revised. We no longer describe the findings as demonstrating a restrictive lung pattern or a confirmed restrictive ventilatory defect.

We now use more cautious wording, including:

“lower spirometric volumes suggestive of possible restrictive physiology”

and:

“true restrictive ventilatory impairment cannot be confirmed without lung-volume assessment, particularly total lung capacity.”

The revised interpretation is that lower FEV1 and FVC were not accompanied by a proportional reduction in FEV1/FVC. This may be compatible with possible restrictive physiology, but it does not establish true restriction.

**Changes made:**

The restrictive interpretation was softened throughout the manuscript, and the keywords, Abstract, Results, Discussion, Conclusion, and figure captions were updated.

**Major Comment 6. Modification of the JBI risk-of-bias tool requires stronger justification**

**Reviewer concern:**

The original eight-item JBI checklist had been modified into seven operational domains.

**Response:**
We agree that modifying a validated appraisal tool could reduce transparency. We therefore discontinued the modified seven-domain approach and reassessed all included studies using the original eight-item Joanna Briggs Institute Critical Appraisal Checklist for Analytical Cross-Sectional Studies without modification.

No checklist items were merged, omitted, weighted, or converted into a numerical score. Each item was rated as Yes, No, Unclear, or Not applicable. Overall appraisal was recorded using the original JBI categories of Include, Exclude, or Seek further information.

The principal concerns involved incomplete identification of confounding factors or insufficiently described strategies for addressing confounding in Anandhalakshmi et al., Singh et al., and Senthilnathan et al. All six studies received an overall appraisal of Include, while item-level limitations were incorporated into the GRADE assessment and interpretation of the findings.

**Changes made:**

The Risk of Bias Assessment sections in the Methods and Results were rewritten. Supplementary Appendix S5 now presents the complete original eight-item JBI assessment for each study.

**Major Comment 7. Search strategy description is incomplete in the main manuscript**

**Reviewer concern:**

The main manuscript did not clarify whether MeSH terms, title/abstract terms, synonyms, spelling variants, and complete database-specific Boolean strategies were used.

**Response:**
We agree and expanded the Information Sources and Search Strategy section. The revised manuscript now explains that the search combined controlled vocabulary terms, including MeSH terms, with free-text title/abstract terms, synonyms, and British/American spelling variants.

A representative PubMed/MEDLINE Boolean search strategy has been added to the main manuscript. Complete database-specific strategies for PubMed/MEDLINE, Scopus, and Web of Science Core Collection are provided in Supplementary Appendix S1.

The revised supplementary search appendix now reports:

- The databases searched;
- The final search date of 25 December 2025;
- The database-specific fields used;
- MeSH and free-text terms;
- Synonyms and spelling variants;
- Boolean operators;
- Truncation where applicable;
- Manual reference-list screening; and
- The search-record counts corresponding to the PRISMA diagram.

**Changes made:**

The Information Sources and Search Strategy section and Supplementary Appendix S1 have been substantially expanded.

**Major Comment 8. PROSPERO timing requires clarification**

**Reviewer concern:**

The manuscript described the review as prospectively registered without reporting the registration date or clarifying the timing relative to the review process.

Response:
We agree and have clarified the registration information. The review protocol was registered in PROSPERO on 27 December 2025 under registration number CRD420251274061.

Because the registration occurred after the final database search on 25 December 2025, we removed the term “prospectively registered” from the manuscript. The revised manuscript reports the registration date and number without making a claim that the registration preceded all review procedures.

**Changes made:**

The PROSPERO Registration section now states:

“The systematic review protocol was registered in the International Prospective Register of Systematic Reviews (PROSPERO) on 27 December 2025. Registration number: CRD420251274061.”

**Minor and Editorial Comments**

**Minor Comment 1. Abstract Methods sentence is grammatically incomplete**

**Response:**
The Abstract Methods section was rewritten to clearly identify the databases, search period, eligibility criteria, and analytical approach.

**The revised wording states:**

“Studies were searched in PubMed/MEDLINE, Scopus, and Web of Science Core Collection from database inception to 25 December 2025. Eligible studies compared spirometric outcomes between adults with type 2 diabetes categorized as having good versus poor glycemic control based on HbA1c. Random-effects meta-analyses were conducted using mean differences.”

**Minor Comment 2. Duplicate sentences are present**

**Response:**
Repeated and redundant sentences in the Introduction, Data Extraction, Study Characteristics, Discussion, and clinical-implication sections were removed or consolidated.

**Minor Comment 3. Section heading should be corrected**

**Response:**
The heading “Result” was corrected to “Results.”

**Minor Comment 4. Capitalization and figure citations require correction**

**Response:**
“PRISMA” is now used consistently. Figure citations and captions were standardized as “Figure 1,” “Figure 2,” “Figure 3,” and “Figure 4.”

**Minor Comment 5. Spelling errors remain**

**Response:**
Spelling errors were corrected throughout the manuscript, including:

- “metanalysis” to “meta-analysis”;
- “Data Availabilitiy” to “Data Availability”; and
- “alveolar-capirally” to “alveolar-capillary.”

**Minor Comment 6. Extra punctuation remains**

**Response:**
Double periods, isolated punctuation, missing spaces between sentences, and other punctuation errors were corrected throughout the manuscript.

**Minor Comment 7. Reference-list formatting is problematic**

**Response:**
The reference list was rechecked against the original source metadata and reformatted consistently. The primary-study citations were corrected and aligned with the six studies included in the meta-analysis.

The Senthilnathan et al. citation was corrected to the primary article used in the analysis, and the statistical software citation was updated to Stata Statistical Software: Release 17.

**Minor Comment 8. ATS/ERS claim requires verification**

**Response:**
We agree that a blanket statement of ATS/ERS compliance should not be made unless explicitly reported by every included study. The wording was therefore softened to state that the included studies used standardized spirometric procedures. We avoided claiming universal ATS/ERS compliance across all studies.

Additional Summary of Statistical Revisions

For transparency, the revised statistical workflow included:

1. Re-extraction of data directly from all six primary studies;
2. Verification of outcome-specific sample sizes;
3. Combination of multiple poor-control HbA1c categories using standard pooled-mean and pooled-standard-deviation formulas;
4. Consistent effect coding as poor glycemic control minus good glycemic control;
5. Use of mean differences in liters for FEV1 and FVC;
6. Harmonization of FEV1/FVC into percentage points and use of mean difference;
7. Reanalysis using Stata 17;
8. Application of DerSimonian–Laird random-effects models; and
9. Regeneration of all forest plots and funnel plots using the corrected datasets.

These revisions resulted in minor numerical changes for FEV1 and FVC and a substantively corrected interpretation of the FEV1/FVC ratio. The updated manuscript, tables, figures, and supplementary files have been made internally consistent.

We sincerely thank the reviewer again for the detailed and constructive comments. We believe that the revisions have substantially strengthened the methodological rigor, transparency, and clinical interpretation of the manuscript.

Sincerely,
**Kadek Surya Atmaja**
on behalf of all authors
