# Supplementary material for: HbA1c and pulmonary function in type 2 diabetes mellitus: A systematic review and meta-analysis
Source: PLoS One. 2026 Aug 13;21(8):e0355628. doi: 10.1371/journal.pone.0355628 (PMC13472463; doi:10.1371/journal.pone.0355628)
Supplement: Peer review history

# Peer review: Decision Letter 0

**DOI:** 10.1371/journal.pone.0355628.r001

**Authors:** George Kuryan

4 Mar 2026

Dear Dr. Atmaja,

Thank you for submitting your manuscript to PLOS ONE. After careful consideration, we feel that it has merit but does not fully meet PLOS ONE’s publication criteria as it currently stands. Therefore, we invite you to submit a revised version of the manuscript that addresses the points raised during the review process.

Please submit your revised manuscript by Apr 18 2026 11:59PM. If you will need more time than this to complete your revisions, please reply to this message or contact the journal office at plosone@plos.org. When you're ready to submit your revision, log on to https://www.editorialmanager.com/pone/ and select the 'Submissions Needing Revision' folder to locate your manuscript file.

- A letter that responds to each point raised by the academic editor and reviewer(s). You should upload this letter as a separate file labeled 'Response to Reviewers'.
- A marked-up copy of your manuscript that highlights changes made to the original version. You should upload this as a separate file labeled 'Revised Manuscript with Track Changes'.
- An unmarked version of your revised paper without tracked changes. You should upload this as a separate file labeled 'Manuscript'.

If applicable, we recommend that you deposit your laboratory protocols in protocols.io to enhance the reproducibility of your results. Protocols.io assigns your protocol its own identifier (DOI) so that it can be cited independently in the future. For instructions see: https://journals.plos.org/plosone/s/submission-guidelines#loc-laboratory-protocols. Additionally, PLOS ONE offers an option for publishing peer-reviewed Lab Protocol articles, which describe protocols hosted on protocols.io. Read more information on sharing protocols at https://plos.org/protocols?utm_medium=editorial-email&utm_source=authorletters&utm_campaign=protocols.

We look forward to receiving your revised manuscript.

Kind regards,

George Kuryan

Academic Editor

PLOS One

Journal Requirements:

When submitting your revision, we need you to address these additional requirements.

1. Please ensure that your manuscript meets PLOS ONE's style requirements, including those for file naming. The PLOS ONE style templates can be found at

https://journals.plos.org/plosone/s/file?id=wjVg/PLOSOne_formatting_sample_main_body.pdf and https://journals.plos.org/plosone/s/file?id=ba62/PLOSOne_formatting_sample_title_authors_affiliations.pdf

2. We note that the grant information you provided in the ‘Funding Information’ and ‘Financial Disclosure’ sections do not match.

When you resubmit, please ensure that you provide the correct grant numbers for the awards you received for your study in the ‘Funding Information’ section.

3. Thank you for stating the following financial disclosure:

[The author(s) received no specific funding for this work.].

At this time, please address the following queries:

a) Please clarify the sources of funding (financial or material support) for your study. List the grants or organizations that supported your study, including funding received from your institution.

b) State what role the funders took in the study. If the funders had no role in your study, please state: “The funders had no role in study design, data collection and analysis, decision to publish, or preparation of the manuscript.”

c) If any authors received a salary from any of your funders, please state which authors and which funders.

d) If you did not receive any funding for this study, please state: “The authors received no specific funding for this work.”

Please include your amended statements within your cover letter; we will change the online submission form on your behalf.

4. Thank you for stating the following in your manuscript:

[This study was funded by institutional support from Warmadewa University]

We note that you have provided funding information that is not currently declared in your Funding Statement. However, funding information should not appear in the Acknowledgments section or other areas of your manuscript. We will only publish funding information present in the Funding Statement section of the online submission form.

Please remove any funding-related text from the manuscript and let us know how you would like to update your Funding Statement. Currently, your Funding Statement reads as follows:

[The author(s) received no specific funding for this work.]

Please include your amended statements within your cover letter; we will change the online submission form on your behalf.

5. Please include a separate caption for each figure in your manuscript.

6. We note you have included a table to which you do not refer in the text of your manuscript. Please ensure that you refer to Table 1 in your text; if accepted, production will need this reference to link the reader to the Table.

7. Please include captions for your Supporting Information files at the end of your manuscript, and update any in-text citations to match accordingly. Please see our Supporting Information guidelines for more information: http://journals.plos.org/plosone/s/supporting-information.

If the reviewer comments include a recommendation to cite specific previously published works, please review and evaluate these publications to determine whether they are relevant and should be cited. There is no requirement to cite these works unless the editor has indicated otherwise.

Please review your reference list to ensure that it is complete and correct. If you have cited papers that have been retracted, please include the rationale for doing so in the manuscript text, or remove these references and replace them with relevant current references. Any changes to the reference list should be mentioned in the rebuttal letter that accompanies your revised manuscript. If you need to cite a retracted article, indicate the article’s retracted status in the References list and also include a citation and full reference for the retraction notice.

[Note: HTML markup is below. Please do not edit.]

Reviewers' comments:

Reviewer's Responses to Questions

**Comments to the Author**

1. Is the manuscript technically sound, and do the data support the conclusions?

Reviewer #1: Partly

**********

2. Has the statistical analysis been performed appropriately and rigorously?

Reviewer #1: Yes

**********

3. Have the authors made all data underlying the findings in their manuscript fully available?

The PLOS Data policy

Reviewer #1: Yes

**********

4. Is the manuscript presented in an intelligible fashion and written in standard English?

Reviewer #1: Yes

**********

Reviewer #1: This manuscript presents a well-conducted and timely systematic review and meta-analysis evaluating the association between glycemic control (HbA1c) and pulmonary function in adults with type 2 diabetes mellitus (T2DM). The topic is clinically relevant, addresses an under-recognized complication of diabetes, and contributes meaningful quantitative evidence supporting the concept of the “diabetic lung.” Overall, the study is methodologically sound, clearly written, and presents consistent findings with minimal statistical heterogeneity. With minor revisions, the manuscript would be suitable for publication.

A. Major Strengths

1. Novelty and Clinical Relevance

This appears to be one of the first meta-analyses to quantitatively synthesize absolute spirometric values (FEV₁, FVC, and FEV₁/FVC) stratified by glycemic control in T2DM.

By focusing on actual volumes rather than percent-predicted values, the authors enhance clinical interpretability, particularly in understanding the magnitude of lung volume loss.

The findings reinforce the emerging paradigm of the lung as a target organ of chronic hyperglycemia, which has implications for routine diabetes care.

2. Methodological Rigor

The review follows PRISMA 2020 guidelines and clearly describes eligibility criteria, data extraction, and analytical methods.

Study selection and data extraction by three independent reviewers using Rayyan strengthens internal validity.

Risk of bias was assessed using a recognized tool (JBI), with transparent justification for domain weighting and overall risk categorization.

Use of a random-effects model is appropriate given clinical heterogeneity, even though statistical heterogeneity was negligible.

3. Consistency and Robustness of Findings

All pooled outcomes demonstrated consistent directionality and magnitude, with I² = 0% across analyses.

The effect sizes (–160 mL for FEV₁ and –250 mL for FVC) are clinically meaningful, particularly when interpreted cumulatively over disease duration.

The moderate certainty rating using GRADE is appropriate and well justified.

4. Biological Plausibility

The discussion provides a coherent mechanistic framework, integrating:

Advanced glycation end-product accumulation

Pulmonary microangiopathy

Chronic systemic inflammation

Oxidative stress

Autonomic neuropathy

This strengthens causal inference despite the observational nature of included studies.

B. Major Limitations and Points for Improvement

1. Search Strategy and Timeframe

The stated search period (“June 2025 to December 2025”) is unconventional and potentially confusing.

Clarification is required: Was this the date range of the search or the publication years searched?

If older studies (e.g., Dennis et al. 2010) were included, the search timeframe should reflect that accurately.

Consider adding the complete search strings (not only in supplementary material but summarized briefly in the main text).

2. Confounding and Adjustment

While the authors appropriately acknowledge residual confounding, this limitation is substantial:

Several included studies did not adequately adjust for age, height, sex, smoking status, BMI, or diabetes duration, all of which strongly influence lung volumes.

The reliance on absolute spirometric values further amplifies this concern.

Although justified, this trade-off should be more explicitly framed as a methodological decision with consequences for interpretation.

3. Interpretation of FEV₁/FVC Findings

The reduction in FEV₁/FVC ratio (SMD –0.26), while statistically significant, is modest.

Traditionally, restrictive lung disease is associated with a preserved or increased FEV₁/FVC ratio.

The manuscript would benefit from a clearer discussion on whether this reflects:

Mixed ventilatory physiology

Small airway involvement

Measurement variability

Or early airflow limitation related to inflammation or autonomic dysfunction

4. Limited Study Number and Geographic Concentration

Only six studies were included, with predominant representation from South Asia.

Generalizability to other populations (e.g., African, European, North American cohorts) may be limited.

This should be more clearly acknowledged in the limitations.

C. Minor Comments and Editorial Suggestions

Language and Style

Minor grammatical and typographical issues are present (e.g., “by, quantifying” in the Introduction).

Occasional sentence length could be reduced for clarity.

Terminology

Use consistent terminology throughout (e.g., “crosssectional” --> “cross-sectional”).

Standardize HbA1c cutoff descriptions to avoid confusion.

**********

what does this mean?). If published, this will include your full peer review and any attached files.

If you choose “no”, your identity will remain anonymous but your review may still be made public.

**Do you want your identity to be public for this peer review?**  For information about this choice, including consent withdrawal, please see our Privacy Policy

Reviewer #1: No

**********

[NOTE: If reviewer comments were submitted as an attachment file, they will be attached to this email and accessible via the submission site. Please log into your account, locate the manuscript record, and check for the action link "View Attachments". If this link does not appear, there are no attachment files.]

To ensure your figures meet our technical requirements, please review our figure guidelines: https://journals.plos.org/plosone/s/figures

You may also use PLOS’s free figure tool, NAAS, to help you prepare publication quality figures: https://journals.plos.org/plosone/s/figures#loc-tools-for-figure-preparation.

NAAS will assess whether your figures meet our technical requirements by comparing each figure against our figure specifications.

# Author comment: Author response to Decision Letter 1

**DOI:** 10.1371/journal.pone.0355628.r002

16 Apr 2026

We sincerely thank the Academic Editor and Reviewer for their careful evaluation of our manuscript and for the constructive comments provided.

In response, we have carefully revised the manuscript and addressed all comments raised in the decision letter. Specifically, we have clarified the search strategy and timeframe, strengthened the discussion of residual confounding and the methodological implications of using absolute spirometric values, expanded the interpretation of the FEV₁/FVC findings, and further elaborated the limitations regarding the small number of included studies and their geographic concentration. We also revised the manuscript for language, grammar, terminology consistency, figure captions, Supporting Information captions, data availability wording, funding/disclosure consistency, and reference accuracy.

A detailed point-by-point response to all comments has been prepared and uploaded as a separate “Response to Reviewers” file. We have also submitted both a marked-up version of the manuscript with tracked changes and a clean revised version.

We are grateful for the opportunity to revise and resubmit our work, and we hope that the revised manuscript will now be suitable for further consideration.

Attachment Submitted filename: Response to Reviewers.docx

# Peer review: Decision Letter 1

**DOI:** 10.1371/journal.pone.0355628.r003

**Authors:** George Kuryan

1 May 2026

Dear Dr. Atmaja,

Thank you for submitting your manuscript to PLOS ONE. After careful consideration, we feel that it has merit but does not fully meet PLOS ONE’s publication criteria as it currently stands. Therefore, we invite you to submit a revised version of the manuscript that addresses the points raised during the review process.

Please submit your revised manuscript by Jun 15 2026 11:59PM. If you will need more time than this to complete your revisions, please reply to this message or contact the journal office at plosone@plos.org. When you're ready to submit your revision, log on to https://www.editorialmanager.com/pone/ and select the 'Submissions Needing Revision' folder to locate your manuscript file.

- A letter that responds to each point raised by the academic editor and reviewer(s). You should upload this letter as a separate file labeled 'Response to Reviewers'.
- A marked-up copy of your manuscript that highlights changes made to the original version. You should upload this as a separate file labeled 'Revised Manuscript with Track Changes'.
- An unmarked version of your revised paper without tracked changes. You should upload this as a separate file labeled 'Manuscript'.

If you would like to make changes to your financial disclosure, please include your updated statement in your cover letter. Guidelines for resubmitting your figure files are available below the reviewer comments at the end of this letter.

If applicable, we recommend that you deposit your laboratory protocols in protocols.io to enhance the reproducibility of your results. Protocols.io assigns your protocol its own identifier (DOI) so that it can be cited independently in the future. For instructions see: https://journals.plos.org/plosone/s/submission-guidelines#loc-laboratory-protocols. Additionally, PLOS ONE offers an option for publishing peer-reviewed Lab Protocol articles, which describe protocols hosted on protocols.io. Read more information on sharing protocols at https://plos.org/protocols?utm_medium=editorial-email&utm_source=authorletters&utm_campaign=protocols.

As the corresponding author, your ORCID iD is verified in the submission system and will appear in the published article. PLOS supports the use of ORCID, and we encourage all coauthors to register for an ORCID iD and use it as well. Please encourage your coauthors to verify their ORCID iD within the submission system before final acceptance, as unverified ORCID iDs will not appear in the published article. *Only*  the individual author can complete the verification step; PLOS staff *cannot*  verify ORCID iDs on behalf of authors.

We look forward to receiving your revised manuscript.

Kind regards,

George Kuryan

Academic Editor

PLOS One

**Journal Requirements:**

1. If the reviewer comments include a recommendation to cite specific previously published works, please review and evaluate these publications to determine whether they are relevant and should be cited. There is no requirement to cite these works unless the editor has indicated otherwise.

2. Please review your reference list to ensure that it is complete and correct. If you have cited papers that have been retracted, please include the rationale for doing so in the manuscript text, or remove these references and replace them with relevant current references. Any changes to the reference list should be mentioned in the rebuttal letter that accompanies your revised manuscript. If you need to cite a retracted article, indicate the article’s retracted status in the References list and also include a citation and full reference for the retraction notice.

[Note: HTML markup is below. Please do not edit.]

Reviewers' comments:

Reviewer's Responses to Questions

**Comments to the Author**

Reviewer #2: All comments have been addressed

**********

2. Is the manuscript technically sound, and do the data support the conclusions?

Reviewer #2: Partly

**********

3. Has the statistical analysis been performed appropriately and rigorously?

Reviewer #2: Yes

**********

4. Have the authors made all data underlying the findings in their manuscript fully available?

The PLOS Data policy

Reviewer #2: Yes

**********

5. Is the manuscript presented in an intelligible fashion and written in standard English?

Reviewer #2: Yes

**********

Reviewer #2: The manuscript highlights an important, yet infrequently discussed and even more poorly understood aspect of diabetes-lung function and glycaemic control. Although the statistical methods and study selection criteria are robustly described, the fact that potential confounding factors such as weight, BMI, race, diet, muscle mass etc have not been considered limit the validity and generalizability of the results; having the same acknowledged as a limitation of the study should suffice in this case.

Attention is drawn to reference no. 7. The authors may want to recheck if this is a valid reference (or has been cited correctly).

**********

what does this mean?). If published, this will include your full peer review and any attached files.

If you choose “no”, your identity will remain anonymous but your review may still be made public.

**Do you want your identity to be public for this peer review?**  For information about this choice, including consent withdrawal, please see our Privacy Policy

Reviewer #2: No

**********

[NOTE: If reviewer comments were submitted as an attachment file, they will be attached to this email and accessible via the submission site. Please log into your account, locate the manuscript record, and check for the action link "View Attachments". If this link does not appear, there are no attachment files.]

To ensure your figures meet our technical requirements, please review our figure guidelines: https://journals.plos.org/plosone/s/figures

You may also use PLOS’s free figure tool, NAAS, to help you prepare publication quality figures: https://journals.plos.org/plosone/s/figures#loc-tools-for-figure-preparation.

NAAS will assess whether your figures meet our technical requirements by comparing each figure against our figure specifications.

# Author comment: Author response to Decision Letter 2

**DOI:** 10.1371/journal.pone.0355628.r004

10 May 2026

Dear Reviewers,

We sincerely thank the Academic Editor and Reviewer #2 for the careful evaluation of our revised manuscript and for the constructive comments that have helped us further improve the clarity, rigor, and interpretability of the manuscript. We are grateful that Reviewer #2 indicated that all previous comments had been addressed, that the statistical analysis was considered appropriate and rigorous, that the data availability was acceptable, and that the manuscript was written in standard English. In this revision, we have addressed the remaining concerns by strengthening the discussion of residual confounding, clarifying the limitations of using absolute spirometric values, tempering causal language throughout the manuscript, correcting reference no. 7, rechecking the full reference list, and correcting the bibliographic details of reference no. 20 based on the original article.

We again thank the Academic Editor and Reviewer #2 for their careful assessment and constructive guidance. We believe the revised manuscript now provides a more cautious and methodologically transparent interpretation of the evidence, explicitly acknowledges the limitations related to residual confounding and generalizability, and contains a corrected and more accurate reference list. We hope that the revised manuscript is now suitable for further consideration by PLOS ONE.

Sincerely,

Kadek Surya Atmaja, MD, Pulmonology Specialist

On behalf of all authors

Attachment Submitted filename: Response_to_Reviewers_2.docx

# Peer review: Decision Letter 2

**DOI:** 10.1371/journal.pone.0355628.r005

**Authors:** George Kuryan

24 May 2026

Dear Dr. Atmaja

Thank you for submitting your manuscript to PLOS ONE. After careful consideration, we feel that it has merit but does not fully meet PLOS ONE’s publication criteria as it currently stands. Therefore, we invite you to submit a revised version of the manuscript that addresses the points raised during the review process.

Please submit your revised manuscript by Jul 08 2026 11:59PM. If you will need more time than this to complete your revisions, please reply to this message or contact the journal office at plosone@plos.org. When you're ready to submit your revision, log on to https://www.editorialmanager.com/pone/ and select the 'Submissions Needing Revision' folder to locate your manuscript file.

- A letter that responds to each point raised by the academic editor and reviewer(s). You should upload this letter as a separate file labeled 'Response to Reviewers'.
- A marked-up copy of your manuscript that highlights changes made to the original version. You should upload this as a separate file labeled 'Revised Manuscript with Track Changes'.
- An unmarked version of your revised paper without tracked changes. You should upload this as a separate file labeled 'Manuscript'.

If you would like to make changes to your financial disclosure, please include your updated statement in your cover letter. Guidelines for resubmitting your figure files are available below the reviewer comments at the end of this letter.

If applicable, we recommend that you deposit your laboratory protocols in protocols.io to enhance the reproducibility of your results. Protocols.io assigns your protocol its own identifier (DOI) so that it can be cited independently in the future. For instructions see: https://journals.plos.org/plosone/s/submission-guidelines#loc-laboratory-protocols. Additionally, PLOS ONE offers an option for publishing peer-reviewed Lab Protocol articles, which describe protocols hosted on protocols.io. Read more information on sharing protocols at https://plos.org/protocols?utm_medium=editorial-email&utm_source=authorletters&utm_campaign=protocols.

As the corresponding author, your ORCID iD is verified in the submission system and will appear in the published article. PLOS supports the use of ORCID, and we encourage all coauthors to register for an ORCID iD and use it as well. Please encourage your coauthors to verify their ORCID iD within the submission system before final acceptance, as unverified ORCID iDs will not appear in the published article. *Only*  the individual author can complete the verification step; PLOS staff *cannot*  verify ORCID iDs on behalf of authors.

We look forward to receiving your revised manuscript.

Kind regards,

George Kuryan

Academic Editor

PLOS One

Journal Requirements:

If the reviewer comments include a recommendation to cite specific previously published works, please review and evaluate these publications to determine whether they are relevant and should be cited. There is no requirement to cite these works unless the editor has indicated otherwise.

[Note: HTML markup is below. Please do not edit.]

Reviewers' comments:

Reviewer's Responses to Questions

**Comments to the Author**

Reviewer #3: All comments have been addressed

Reviewer #4: All comments have been addressed

**********

2. Is the manuscript technically sound, and do the data support the conclusions?

Reviewer #3: Yes

Reviewer #4: Yes

**********

3. Has the statistical analysis been performed appropriately and rigorously?

Reviewer #3: Yes

Reviewer #4: Yes

**********

4. Have the authors made all data underlying the findings in their manuscript fully available?

The PLOS Data policy

Reviewer #3: Yes

Reviewer #4: Yes

**********

5. Is the manuscript presented in an intelligible fashion and written in standard English?

Reviewer #3: Yes

Reviewer #4: Yes

**********

Reviewer #3: Reviewer Assessment

HbA1c and Pulmonary Function in Type 2 Diabetes Mellitus: A Systematic Review and Meta-Analysis

Journal / Platform PLOS ONE

Manuscript number PONE-D-25-68675R2

Article type Research Article

Overall recommendation Major revision before acceptance

Overall assessment

This systematic review and meta-analysis evaluate the association between poor glycemic control, measured by HbA1c, and pulmonary function in adults with type 2 diabetes mellitus. The topic is appropriate and clinically meaningful. The overall direction of findings is consistent, with lower FEV1 and FVC among individuals with poorer glycemic control. Nevertheless, the manuscript still contains internal inconsistencies and methodological overstatements that must be addressed.

Major comments

1. PRISMA flow numbers are internally inconsistent

Reviewer concern: The study selection section reports 1,243 records identified, 948 records screened after duplicate removal, 901 records excluded at title/abstract screening, 44 full-text articles assessed, 41 full-text articles excluded, and 6 studies included. These numbers do not align. If 948 records were screened and 901 were excluded, then 47 full-text articles should have been assessed, not 44. If 44 full-text articles were assessed and 41 were excluded, then only 3 studies should remain, not 6. This is a major reporting problem and must be corrected in both the text and the PRISMA figure.

Required revision: Please revise the PRISMA flow diagram and study selection text so that all numbers are internally consistent.

2. Inconsistency in the number of studies included for FEV1/FVC ratio

Reviewer concern: The Study Characteristics section states that four studies provided FEV1/FVC data, but the Results section states that five studies contributed data to the FEV1/FVC ratio meta-analysis. The table appears to show FEV1/FVC data for Anandhalakshmi, Maan, Singh, and Senthilnathan only, whereas Barik and Dennis are listed as providing only FEV1 and FVC. This discrepancy directly affects the validity of the FEV1/FVC pooled estimate.

Required revision: Please clearly state the exact number of studies and participants contributing to each pooled outcome: FEV1, FVC, and FEV1/FVC.

3. GRADE rating of “moderate certainty” may be overstated

Reviewer concern: The manuscript states that all included studies were observational, so the initial certainty of evidence was low, but later concludes that the certainty of evidence was moderate for all outcomes. This needs stronger justification. Several included studies had incomplete adjustment for key confounders such as age, sex, BMI, smoking status, height, ethnicity, diabetes duration, physical activity, nutritional status, and muscle mass. The manuscript itself acknowledges residual confounding as a major limitation.

Required revision: Please either downgrade the certainty to low or provide a clear GRADE justification for upgrading from low to moderate, such as large effect size, dose-response gradient, or residual confounding that would likely reduce rather than increase the observed association.

4. Use of absolute spirometric values limits interpretability

Reviewer concern: The manuscript includes only absolute spirometric values in liters. This improves direct pooling but limits clinical interpretation because FEV1 and FVC are strongly influenced by age, sex, height, ethnicity, and body size. This limitation is mentioned but should be emphasized more strongly because it directly affects cross-study comparability and interpretation of clinical significance.

Required revision: Please avoid implying that the observed absolute reductions necessarily represent clinically meaningful impairment without percent-predicted values, lower limit of normal, or total lung capacity.

5. The FEV1/FVC finding conflicts with a purely restrictive interpretation

Reviewer concern: The manuscript suggests a restrictive pattern because both FEV1 and FVC are reduced. However, the pooled FEV1/FVC ratio is also reported as reduced. Classical restrictive ventilatory impairment usually has a preserved or increased FEV1/FVC ratio. Although the authors acknowledge this later, the abstract and conclusion still present the findings too strongly as a restrictive lung pattern.

Required revision: Please soften “restrictive lung pattern” throughout the manuscript. Prefer phrases such as “reduced spirometric volumes suggestive of possible restrictive physiology” or “reduced lung volumes without definitive confirmation of restriction.” True restriction requires lung volume measurement, particularly total lung capacity.

6. Modification of the JBI risk-of-bias tool requires stronger justification

Reviewer concern: The manuscript states that the original JBI checklist has eight items, but two items were combined because they were conceptually overlapping, resulting in seven domains. Modifying a validated tool can be questioned unless the process is transparent.

Required revision: Please specify exactly which two JBI items were combined and provide both the original eight-item assessment and the modified seven-domain reconciliation in the supplement.

7. Search strategy description is incomplete in the main manuscript

Reviewer concern: The manuscript describes the search terms broadly and refers readers to the supplement for the detailed strategy. For reproducibility, the main Methods section should briefly clarify whether MeSH terms, title/abstract terms, synonyms, and spelling variants were used.

Required revision: Please include at least one representative search string in the manuscript or ensure the supplementary file provides complete database-specific Boolean strategies.

8. PROSPERO timing requires clarification

Reviewer concern: The manuscript states that the protocol was prospectively registered in PROSPERO, but it does not clearly state the date of registration or whether registration occurred before screening and data extraction were completed.

Required revision: Please add the date of PROSPERO registration and clarify whether registration occurred before completion of study selection and data extraction. If registration occurred after screening began, the wording “prospectively registered” should be modified.

Suggested exact wording for key revisions

For absolute spirometric values

Suggested wording:

Because spirometric values were pooled as absolute volumes rather than percent-predicted values, the observed mean differences should be interpreted as group-level associations rather than definitive evidence of clinically abnormal lung function.

For clinical implication

Suggested wording:

These findings support awareness of possible pulmonary involvement in patients with poorly controlled or long-standing type 2 diabetes, particularly when respiratory symptoms or additional cardiometabolic risk factors are present.

For restrictive interpretation

Suggested wording:

The reductions in FEV1 and FVC suggest lower spirometric volumes among individuals with poor glycemic control; however, confirmation of true restrictive ventilatory impairment requires lung volume assessment, particularly total lung capacity.

Minor and editorial comments

1. Abstract Methods sentence is grammatically incomplete. Revise to: “We searched PubMed, Scopus, and Web of Science from database inception to 25 December 2025. Studies were eligible if they compared spirometric outcomes between adults with type 2 diabetes categorized by good versus poor glycemic control based on HbA1c.”

2. Duplicate sentences are present. Remove repeated sentences in the Introduction, Data Extraction, and Study Characteristics sections.

3. Section heading should be corrected. Change “Result” to “Results.”

4. Capitalization and figure citation need correction. Use “PRISMA” consistently and write “Figure 1” rather than “fig 1.”

5. Spelling errors remain. Correct “metanalysis” to “meta-analysis,” “Data Availabilitiy” to “Data Availability,” and “alveolar-capirally” to “alveolar-capillary.”

6. Extra punctuation remains. Remove double periods such as “duration..”, “study..”, and “reporting of this study..”.

7. Reference list formatting is problematic. References 6–9 appear disrupted, with numbering and author details broken across lines. Recheck the full reference list against journal style and original source metadata.

8. ATS/ERS claim requires verification. State this only if each included study explicitly reported ATS/ERS-compliant spirometry. Otherwise soften to “Most included studies reported standardized spirometric assessment; where available, spirometry was performed according to ATS/ERS-based procedures.”

Outcome-specific clarification table requested from authors

Outcome Number of studies Total participants

FEV1 6 To be stated

FVC 6 To be stated

FEV1/FVC ratio Clarify: 4 or 5 To be stated

Final reviewer recommendation

Recommendation: Major revision

Reviewer #4: Revision: Temporal limitation of the Biomarker (HbA1c)

The authors cited a long-term structural mechanism, such as extracellular matrix glycation and pulmonary microangiopathy to justify why the lung function drops. However, while the structural tissue remodeling takes years of chronic hyperglycemia to develop, HBA1c is a short-term biomarker reflecting glycaemic status over only the preceding 8-12 weeks.

The authors are requested to address this temporal mismatch:

1. Please add a brief discussion to the limitation section noting that relying on a short-term baseline biomarker to explain chronic, structural organ damage is a key limitation, especially given the cross-sectional nature of the underlying date.

2. Please comment on how potential patient misclassification might affect your pooled mean differences. For example, if a patient with uncontrolled diabetes (including lung changes) for ten years, who achieved strict glycemic control only in the 3 months preceding the study would be misclassified into the “good control” cohort, potentially blurring or underestimating the true impact of chronic hyperglycemia.

**********

what does this mean?). If published, this will include your full peer review and any attached files.

If you choose “no”, your identity will remain anonymous but your review may still be made public.

**Do you want your identity to be public for this peer review?**  For information about this choice, including consent withdrawal, please see our Privacy Policy

Reviewer #3: **Yes:** Vivek Jha

Reviewer #4: No

**********

[NOTE: If reviewer comments were submitted as an attachment file, they will be attached to this email and accessible via the submission site. Please log into your account, locate the manuscript record, and check for the action link "View Attachments". If this link does not appear, there are no attachment files.]

To ensure your figures meet our technical requirements, please review our figure guidelines: https://journals.plos.org/plosone/s/figures

You may also use PLOS’s free figure tool, NAAS, to help you prepare publication quality figures: https://journals.plos.org/plosone/s/figures#loc-tools-for-figure-preparation.

NAAS will assess whether your figures meet our technical requirements by comparing each figure against our figure specifications.

Attachment Submitted filename: PFT IN DM.docx

Attachment Submitted filename: Review.docx

# Author comment: Author response to Decision Letter 3

**DOI:** 10.1371/journal.pone.0355628.r006

26 Jun 2026

Dear Editor and Reviewer,

We sincerely thank the reviewer for the careful and constructive evaluation of our manuscript. The comments were highly valuable and helped us improve the internal consistency, methodological transparency, statistical analysis, and clinical interpretation of the review.

In response, we rechecked the study-selection records, re-extracted the outcome data directly from the six primary studies, reran all meta-analyses using Stata, revised the certainty-of-evidence assessment, returned to the original unmodified eight-item JBI checklist, expanded the search-strategy description, clarified the PROSPERO registration date, and revised the interpretation of the spirometric findings throughout the manuscript.

Our point-by-point responses are provided below.

________________________________________

Major Comment 1. PRISMA flow numbers are internally inconsistent

Reviewer concern:

The numbers reported for records screened, full-text reports assessed, full-text exclusions, and included studies were internally inconsistent.

Response:

We agree with the reviewer. We rechecked the study-selection records and revised both the PRISMA 2020 flow diagram and the Study Selection section so that all numbers are internally consistent.

The corrected study-selection flow is:

• Records identified: 1,243

• Duplicate records removed: 295

• Records screened: 948

• Records excluded at title/abstract screening: 901

• Reports sought for retrieval: 47

• Reports not retrieved: 3

• Full-text reports assessed for eligibility: 44

• Full-text reports excluded: 38

• Studies included in the quantitative synthesis: 6

The 38 full-text exclusions comprised incomplete reporting of spirometric data (n = 12), absence of glycemic-control categories (n = 15), and inappropriate study design (n = 11). Thus, 44 minus 38 correctly results in six included studies.

Changes made:

The Study Selection section and Figure 1 have been revised using the corrected PRISMA numbers.

________________________________________

Major Comment 2. Inconsistency in the number of studies included for the FEV1/FVC ratio

Reviewer concern:

The manuscript inconsistently reported four or five studies contributing to the FEV1/FVC meta-analysis, and the study-characteristics table did not clearly identify all contributing studies.

Response:

We agree and have corrected this discrepancy. We re-extracted outcome-specific data directly from the six primary studies and clearly stated the exact number of studies and participants contributing to each pooled outcome:

• FEV1: 6 studies, 944 participants

• FVC: 6 studies, 944 participants

• FEV1/FVC ratio: 5 studies, 801 participants

The five studies contributing to the FEV1/FVC analysis were Anandhalakshmi et al., Maan et al., Singh et al., Senthilnathan et al., and Dennis et al. Barik et al. was not included in the FEV1/FVC meta-analysis because extractable group-specific mean and standard deviation values for the ratio were not available.

Table 1 was revised to show that Dennis et al. contributed FEV1, FVC, and FEV1/FVC data. Table 2 was added to explicitly present the number of studies, participants, and study names contributing to each pooled outcome.

To fully resolve the inconsistency, we re-extracted all spirometric data directly from the six primary studies rather than relying on the previously compiled manuscript dataset. Several studies reported more than two HbA1c categories. For these studies, categories above the study-specific good-control threshold were combined into a single poor-control group using standard formulas for pooled means and pooled standard deviations.

All meta-analyses were then rerun in Stata 17. Stata was selected because it enabled a transparent and reproducible workflow for:

1. Combining multiple HbA1c categories before meta-analysis;

2. Verifying study-specific sample sizes, means, and standard deviations;

3. Applying a uniform effect direction defined as poor glycemic control minus good glycemic control;

4. Harmonizing FEV1/FVC values into percentage points;

5. Generating outcome-specific forest plots and funnel plots; and

6. Maintaining a reproducible analytical dataset and command syntax.

FEV1 and FVC were pooled as mean differences in liters. Because the FEV1/FVC ratio represents the same clinical measure across studies and could be harmonized into percentage points, it was pooled using a mean difference rather than a standardized mean difference. This preserves clinical interpretability.

The revised pooled results were:

• FEV1: MD = −0.16 L, 95% CI −0.25 to −0.07; p < 0.001; I² = 0%

• FVC: MD = −0.24 L, 95% CI −0.35 to −0.14; p < 0.001; I² = 0%

• FEV1/FVC ratio: MD = 1.61 percentage points, 95% CI 0.50 to 2.73; p = 0.005; I² = 0%

The numerical changes were primarily caused by corrected study-level data extraction, consistent outcome coding, category harmonization, and use of the clinically appropriate effect measure.

Changes made:

The Study Characteristics section, Tables 1 and 2, Statistical Analysis section, Results, Abstract, Discussion, forest plots, funnel plots, and figure captions have been revised accordingly.

________________________________________

Major Comment 3. GRADE rating of “moderate certainty” may be overstated

Reviewer concern:

All included studies were observational, and there was insufficient justification for upgrading the certainty of evidence from low to moderate.

Response:

We agree. The GRADE assessment has been revised, and the certainty of evidence for FEV1, FVC, and FEV1/FVC is now rated as low.

All outcomes started at low certainty because the included studies were observational. No outcome was upgraded because there was no prespecified evidence of a large effect, a dose-response gradient, or residual confounding that would be expected to increase the observed association.

Although statistical heterogeneity was negligible and the pooled confidence intervals excluded the null effect, several studies incompletely identified or adjusted for important confounders, including age, sex, height, body size or BMI, smoking status, ethnicity, diabetes duration, physical activity, nutritional status, and muscle mass.

Changes made:

The Certainty of Evidence Assessment section and Supplementary Appendix S3 have been revised. All three outcomes are now classified as low-certainty evidence.

________________________________________

Major Comment 4. Use of absolute spirometric values limits interpretability

Reviewer concern:

Absolute FEV1 and FVC values are influenced by age, sex, height, ethnicity, and body size and therefore cannot be interpreted as definitive evidence of clinically abnormal lung function.

Response:

We agree and have strengthened this limitation throughout the manuscript. FEV1 and FVC were pooled as absolute volumes in liters because group-specific percent-predicted values, z-scores, or lower-limit-of-normal classifications were not consistently available across the included studies.

We now explicitly state that the pooled mean differences represent group-level associations rather than definitive evidence of clinically abnormal pulmonary function. We also clarify that residual demographic and anthropometric differences may have influenced the estimates.

We avoided interpreting the observed reductions as necessarily clinically meaningful impairment and emphasized the need for future studies to report percent-predicted values, z-scores, lower-limit-of-normal classifications, and lung-volume measurements.

Changes made:

The Abstract, Results, Discussion, Strengths and Limitations, and Conclusion have been revised to emphasize the limitations of absolute spirometric values.

________________________________________

Major Comment 5. The FEV1/FVC finding conflicts with a purely restrictive interpretation

Reviewer concern:

A restrictive ventilatory impairment generally has a preserved or increased FEV1/FVC ratio, whereas the previous manuscript reported a reduced pooled ratio and nevertheless interpreted the findings as restrictive.

Response:

We agree. Following direct re-extraction and reanalysis of the primary-study data, the FEV1/FVC ratio was not reduced. The revised analysis showed a small but statistically significant increase in the ratio among participants with poor glycemic control:

MD = 1.61 percentage points, 95% CI 0.50 to 2.73; p = 0.005.

The Abstract, Results, Discussion, and Conclusion have therefore been revised. We no longer describe the findings as demonstrating a restrictive lung pattern or a confirmed restrictive ventilatory defect.

We now use more cautious wording, including:

“lower spirometric volumes suggestive of possible restrictive physiology”

and:

“true restrictive ventilatory impairment cannot be confirmed without lung-volume assessment, particularly total lung capacity.”

The revised interpretation is that lower FEV1 and FVC were not accompanied by a proportional reduction in FEV1/FVC. This may be compatible with possible restrictive physiology, but it does not establish true restriction.

Changes made:

The restrictive interpretation was softened throughout the manuscript, and the keywords, Abstract, Results, Discussion, Conclusion, and figure captions were updated.

________________________________________

Major Comment 6. Modification of the JBI risk-of-bias tool requires stronger justification

Reviewer concern:

The original eight-item JBI checklist had been modified into seven operational domains.

Response:

We agree that modifying a validated appraisal tool could reduce transparency. We therefore discontinued the modified seven-domain approach and reassessed all included studies using the original eight-item Joanna Briggs Institute Critical Appraisal Checklist for Analytical Cross-Sectional Studies without modification.

No checklist items were merged, omitted, weighted, or converted into a numerical score. Each item was rated as Yes, No, Unclear, or Not applicable. Overall appraisal was recorded using the original JBI categories of Include, Exclude, or Seek further information.

The principal concerns involved incomplete identification of confounding factors or insufficiently described strategies for addressing confounding in Anandhalakshmi et al., Singh et al., and Senthilnathan et al. All six studies received an overall appraisal of Include, while item-level limitations were incorporated into the GRADE assessment and interpretation of the findings.

Changes made:

The Risk of Bias Assessment sections in the Methods and Results were rewritten. Supplementary Appendix S5 now presents the complete original eight-item JBI assessment for each study.

________________________________________

Major Comment 7. Search strategy description is incomplete in the main manuscript

Reviewer concern:

The main manuscript did not clarify whether MeSH terms, title/abstract terms, synonyms, spelling variants, and complete database-specific Boolean strategies were used.

Response:

We agree and expanded the Information Sources and Search Strategy section. The revised manuscript now explains that the search combined controlled vocabulary terms, including MeSH terms, with free-text title/abstract terms, synonyms, and British/American spelling variants.

A representative PubMed/MEDLINE Boolean search strategy has been added to the main manuscript. Complete database-specific strategies for PubMed/MEDLINE, Scopus, and Web of Science Core Collection are provided in Supplementary Appendix S1.

The revised supplementary search appendix now reports:

• The databases searched;

• The final search date of 25 December 2025;

• The database-specific fields used;

• MeSH and free-text terms;

• Synonyms and spelling variants;

• Boolean operators;

• Truncation where applicable;

• Manual reference-list screening; and

• The search-record counts corresponding to the PRISMA diagram.

Changes made:

The Information Sources and Search Strategy section and Supplementary Appendix S1 have been substantially expanded.

________________________________________

Major Comment 8. PROSPERO timing requires clarification

Reviewer concern:

The manuscript described the review as prospectively registered without reporting the registration date or clarifying the timing relative to the review process.

Response:

We agree and have clarified the registration information. The review protocol was registered in PROSPERO on 27 December 2025 under registration number CRD420251274061.

Because the registration occurred after the final database search on 25 December 2025, we removed the term “prospectively registered” from the manuscript. The revised manuscript reports the registration date and number without making a claim that the registration preceded all review procedures.

Changes made:

The PROSPERO Registration section now states:

“The systematic review protocol was registered in the International Prospective Register of Systematic Reviews (PROSPERO) on 27 December 2025. Registration number: CRD420251274061.”

________________________________________

Minor and Editorial Comments

Minor Comment 1. Abstract Methods sentence is grammatically incomplete

Response:

The Abstract Methods section was rewritten to clearly identify the databases, search period, eligibility criteria, and analytical approach.

The revised wording states:

“Studies were searched in PubMed/MEDLINE, Scopus, and Web of Science Core Collection from database inception to 25 December 2025. Eligible studies compared spirometric outcomes between adults with type 2 diabetes categorized as having good versus poor glycemic control based on HbA1c. Random-effects meta-analyses were conducted using mean differences.”

________________________________________

Minor Comment 2. Duplicate sentences are present

Response:

Repeated and redundant sentences in the Introduction, Data Extraction, Study Characteristics, Discussion, and clinical-implication sections were removed or consolidated.

________________________________________

Minor Comment 3. Section heading should be corrected

Response:

The heading “Result” was corrected to “Results.”

________________________________________

Minor Comment 4. Capitalization and figure citations require correction

Response:

“PRISMA” is now used consistently. Figure citations and captions were standardized as “Figure 1,” “Figure 2,” “Figure 3,” and “Figure 4.”

________________________________________

Minor Comment 5. Spelling errors remain

Response:

Spelling errors were corrected throughout the manuscript, including:

• “metanalysis” to “meta-analysis”;

• “Data Availabilitiy” to “Data Availability”; and

• “alveolar-capirally” to “alveolar-capillary.”

________________________________________

Minor Comment 6. Extra punctuation remains

Response:

Double periods, isolated punctuation, missing spaces between sentences, and other punctuation errors were corrected throughout the manuscript.

________________________________________

Minor Comment 7. Reference-list formatting is problematic

Response:

The reference list was rechecked against the original source metadata and reformatted consistently. The primary-study citations were corrected and aligned with the six studies included in the meta-analysis.

The Senthilnathan et al. citation was corrected to the primary article used in the analysis, and the statistical software citation was updated to Stata Statistical Software: Release 17.

________________________________________

Minor Comment 8. ATS/ERS claim requires verification

Response:

We agree that a blanket statement of ATS/ERS compliance should not be made unless explicitly reported by every included study. The wording was therefore softened to state that the included studies used standardized spirometric procedures. We avoided claiming universal ATS/ERS compliance across all studies.

________________________________________

Additional Summary of Statistical Revisions

For transparency, the revised statistical workflow included:

1. Re-extraction of data directly from all six primary studies;

2. Verification of outcome-specific sample sizes;

3. Combination of multiple poor-control HbA1c categories using standard pooled-mean and pooled-standard-deviation formulas;

4. Consistent effect coding as poor glycemic control minus

Attachment Submitted filename: Response_to_Reviewers_auresp_3.docx

# Peer review: Decision Letter 3

**DOI:** 10.1371/journal.pone.0355628.r007

**Authors:** George Kuryan

23 Jul 2026

<p>HbA1c and Pulmonary Function in Type 2 Diabetes Mellitus: A Systematic Review and Meta-Analysis

PONE-D-25-68675R3

Dear Dr. Atmaja,

We’re pleased to inform you that your manuscript has been judged scientifically suitable for publication and will be formally accepted for publication once it meets all outstanding technical requirements.

Within one week, you’ll receive an e-mail detailing the required amendments. When these have been addressed, you’ll receive a formal acceptance letter and your manuscript will be scheduled for publication.

An invoice will be generated when your article is formally accepted. Please note, if your institution has a publishing partnership with PLOS and your article meets the relevant criteria, all or part of your publication costs will be covered. Please make sure your user information is up-to-date by logging into Editorial Manager at Editorial Manager® and clicking the ‘Update My Information' link at the top of the page. For questions related to billing, please contact billing support.

If your institution or institutions have a press office, please notify them about your upcoming paper to help maximize its impact. If they’ll be preparing press materials, please inform our press team as soon as possible -- no later than 48 hours after receiving the formal acceptance. Your manuscript will remain under strict press embargo until 2 pm Eastern Time on the date of publication. For more information, please contact onepress@plos.org.

Kind regards,

George Kuryan

Academic Editor

PLOS One

Additional Editor Comments (optional):

Reviewers' comments:

Reviewer's Responses to Questions

**Comments to the Author**

Reviewer #5: All comments have been addressed

**********

2. Is the manuscript technically sound, and do the data support the conclusions?

Reviewer #5: Yes

**********

3. Has the statistical analysis been performed appropriately and rigorously?

Reviewer #5: Yes

**********

4. Have the authors made all data underlying the findings in their manuscript fully available?

The PLOS Data policy

Reviewer #5: Yes

**********

5. Is the manuscript presented in an intelligible fashion and written in standard English?

Reviewer #5: Yes

**********

Reviewer #5: All the comments are well addressed and mentioned clearly by the author. The only concern is that the studies available are limited to six; otherwise, the author has replied to the queries in a structured manner.

**********

what does this mean?). If published, this will include your full peer review and any attached files.

If you choose “no”, your identity will remain anonymous but your review may still be made public.

**Do you want your identity to be public for this peer review?**  For information about this choice, including consent withdrawal, please see our Privacy Policy

Reviewer #5: **Yes:** KISHORE KUMAR BEHERA,

**********

# Editor report: Acceptance letter

**DOI:** 10.1371/journal.pone.0355628.r008

**Authors:** George Kuryan

PONE-D-25-68675R3

PLOS One

Dear Dr. Atmaja,

I'm pleased to inform you that your manuscript has been deemed suitable for publication in PLOS One. Congratulations! Your manuscript is now being handed over to our production team.

At this stage, our production department will prepare your paper for publication. This includes ensuring the following:

* All references, tables, and figures are properly cited

* All relevant supporting information is included in the manuscript submission,

* There are no issues that prevent the paper from being properly typeset

You will receive further instructions from the production team, including instructions on how to review your proof when it is ready. Please keep in mind that we are working through a large volume of accepted articles, so please give us a few days to review your paper and let you know the next and final steps.

Lastly, if your institution or institutions have a press office, please let them know about your upcoming paper now to help maximize its impact. If they'll be preparing press materials, please inform our press team within the next 48 hours. Your manuscript will remain under strict press embargo until 2 pm Eastern Time on the date of publication. For more information, please contact onepress@plos.org.

You will receive an invoice from PLOS for your publication fee after your manuscript has reached the completed accept phase. If you receive an email requesting payment before acceptance or for any other service, this may be a phishing scheme. Learn how to identify phishing emails and protect your accounts at https://explore.plos.org/phishing.

If we can help with anything else, please email us at customercare@plos.org.

Thank you for submitting your work to PLOS ONE and supporting open access.

Kind regards,

PLOS ONE Editorial Office Staff

on behalf of

Professor George Kuryan

Academic Editor

PLOS One
